# Substrate engagement of integrins α_5_β_1_ and α_v_β_3_ is necessary, but not sufficient, for high directional persistence in migration on fibronectin

**DOI:** 10.1038/srep23258

**Published:** 2016-03-18

**Authors:** Dimitris Missirlis, Tamás Haraszti, Catharina v. C. Scheele, Tina Wiegand, Carolina Diaz, Stefanie Neubauer, Florian Rechenmacher, Horst Kessler, Joachim P. Spatz

**Affiliations:** 1 Department of New Materials and Biosystems, Max Planck Institute for Intelligent Systems & University of Heidelberg, Department of Biophysical Chemistry Heisenbergstr. 3, D-70569 Stuttgart, Germany; 2 Institute for Advanced Study (IAS) and Center of Integrated Protein Science (CIPSM), Department Chemie, Technische Universität München, Lichtenbergstr. 4, Garching, D-85747, Germany

## Abstract

The interplay between specific integrin-mediated matrix adhesion and directional persistence in cell migration is not well understood. Here, we characterized fibroblast adhesion and migration on the extracellular matrix glycoproteins fibronectin and vitronectin, focusing on the role of α_5_β_1_ and α_v_β_3_ integrins. Fibroblasts manifested high directional persistence in migration on fibronectin-, but not vitronectin-coated substrates, in a ligand density-dependent manner. Fibronectin stimulated α_5_β_1_-dependent organization of the actin cytoskeleton into oriented, ventral stress fibers, and assembly of dynamic, polarized protrusions, characterized as regions free of stress fibers and rich in nascent adhesions at their edge. Such protrusions correlated with persistent, local leading edge advancement, but were not sufficient, nor necessary for directional migration over longer times. Selective blocking of α_v_β_3_ or α_5_β_1_ integrins using small molecule integrin antagonists reduced directional persistence on fibronectin, indicating integrin cooperativity in maintaining directionality. On the other hand, patterned substrates, designed to selectively engage either integrin, or their combination, were not sufficient to establish directional migration. Overall, our study demonstrates adhesive coating-dependent regulation of directional persistence in fibroblast migration and challenges the generality of the previously suggested role of β_1_ and β_3_ integrins in directional migration.

Mesenchymal cell migration involves a complex, yet tightly regulated control over actin polymerization, adhesion dynamics and actomyosin contractility to enable cell translocation in its environment. Much of our understanding on how signals from the extracellular matrix (ECM) control cell migration stems from *in vitro* studies on flat substrates, on which both soluble and insoluble biochemical signals can be precisely manipulated^1^,^2^. Cell adhesion can be modulated by coating with ECM proteins, their fragments or small molecular ligands (e.g. peptides) and by employing engineering strategies to precisely vary ligand presentation, concentrations and mechanics^3^.

Integrins are the major trans-membrane receptors cells employ to recognize, adhere and adapt to the chemical and mechanical properites of their ECM^4^. The 18 α and 8 β subunits assemble into 24 heterodimeric integrin complexes that exhibit varying affinity for ECM ligands and distinct signaling capabilities^5^,^6^. Interestingly, integrin expression profiles are often altered in pathological situations such as during wound healing, angiogenesis or tumor metastasis, presumably to promote efficient cell migration^7^,^8^. While integrins are probably not the sole receptor family responsible in regulating cell migration, understanding how cells respond to differential integrin engagement in respect to their motility, and in particular their directional persistence is a major open question^9^,^10^, and constitutes the underlying motivation of this study.

Among integrins, particular attention has been placed on the “fibronectin receptor” α_5_β_1_ and “vitronectin receptor” α_v_β_3_, and their impact on cell migration^11^. Previous work, based on exogenous integrin expression on cells that originally lack these integrins, has suggested that β_1_ promotes random cell migration, while β_3_ favor persistent migration^12^. More recently, pan-integrin-null fibroblasts were used to show that expression of α_v_ integrins results in increased persistence compared to β_1_ integrin expression, and that there is substantial cross-talk between the two integrin classes^13^. Indeed, employing highly selective integrin peptidomimetics on spatially patterned surfaces, we recently provided further support of integrin cross-talk and demonstrated that integrin α_v_β_3_ co-localizes with integrin α_5_β_1_ also in absence of α_v_β_3_ ligand presentation^14^. The integrin dependence in directional migration was traced to the differential regulation of the family of RhoGTPases and the balance of actin polymerization mediators, including cofilin^12^,^15^. However, the aforementioned studies examining directional migration utilized exogenous control over integrin expression and tested migration only on fibronectin as the cell adhesive coating.

Here, we presented fibroblasts with substrates coated with plasma fibronectin (FN) or vitronectin (VN), both ECM glycoproteins containing the integrin-binding RGD sequence^16^,^17^. In this manner, we studied how differential ECM receptor engagement affects single cell adhesion and migration avoiding genetic manipulation of cells. FN is a major constituent of provisional matrix during wound healing and is the most commonly-used cell adhesive coating for *in vitro* fibroblast migration studies. VN has received less attention, despite being an abundant serum protein, which is adsorbed readily on surfaces *in vitro*^18^, and having exhibited distinct behavior in initial cell motility studies^19^. FN and VN present binding sites for a range of different integrins and membrane receptors, including the heparan sulfate proteoglycan syndecan-4 for FN^20^, and the urokinase plasminogen receptor for VN^21^. Nevertheless, for the reasons outlined above, we focused on the role of α_5_β_1_ as the major integrin receptor for FN, which does not recognize VN, and α_v_β_3_ that can bind both FN and VN^5^.

Our findings revealed a pronounced effect of ECM protein coating on cell motility, with fibroblasts exhibiting directionally persistent migration on FN-coated substrates, as a function of FN surface density. Characterization of fibroblast adhesion and dynamics identified marked differences in adhesion plaque formation, cytoskeleton organization, focal adhesion dynamics and intracellular signaling between FN and VN. In order to examine the role of α_5_β_1_ or α_v_β_3_ integrins, we used highly selective, antagonists against these integrins, either in soluble form to block their substrate engagement, or immobilized on a patterned substrate as cell adhesive ligands. Our results demonstrate that α_5_β_1_ and α_v_β_3_ integrins are necessary but not sufficient for directional persistence in fibroblast migration.

## Results

### High directional persistence in fibroblast migration on fibronectin

Rat embryonic fibroblast (REF_WT_) migration was examined in absence of soluble or insoluble gradients on FN-coated versus VN-coated tissue culture polystyrene (TCPS) as a function of protein coating concentration. FN forms saturated monolayers on TCPS at approximately 10 μg/ml coating concentration^22^, which was confirmed here using an indirect ELISA assay (Supplementary Fig. S1). VN forms monolayers at even lower coating concentrations^23^; indeed, we observed surface saturation at coating concentrations below 1 μg/ml (Supplementary Fig. S1). We excluded the possibility that FN or VN from the cell culture medium adsorbs on the coated substrates (10 μg/ml coating concentration), in order to attribute adhesion and motility to the coated ligands (Supplementary Fig. S1). Moreover, REF_WT_ adhered with similar efficiency on FN and VN, at coating concentrations of 1 and 10 μg/ml, excluding the possibility that we select a population based on differential cell adhesion (Supplementary Fig. S1).

**Figure 1:**
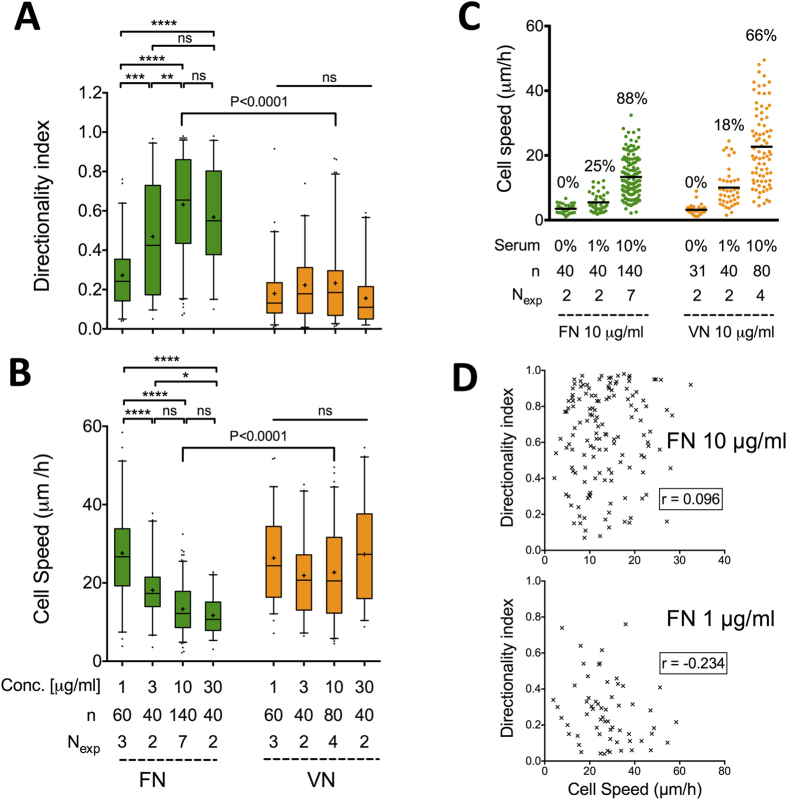
Fibroblasts migrate persistently on FN but not on VN. (**A**) REF_WT_ directionality index (DI) and (**B**) cell speed as a function of FN or VN coating concentration on TCPS-coated substrates. DI equals the ratio of the final distance a cell moved from the origin to the total trajectory length. The middle line in box plots indicates the median, the box indicates the interquartile range, the whiskers the 5^th^ and 95^th^ percentiles and the cross the mean. Data for each coating were analyzed using one-way ANOVA with Tukey post-test analysis: ns: not significant; *P < 0.05, **P < 0.01; ***P < 0.001; ****P < 0.0001. Data for 10 μg/ml FN and VN coating concentration were compared using an unpaired t-test. (**C**) Serum was required for stimulating REF_WT_ migration as indicated by the low cell speed and percentage of motile cells (indicated by % on the graph) in presence of 1% serum or absence of serum in the culture medium. Black lines in dot plot represent mean values. (**D**) No correlation was observed between DI and cell speed for REF_WT_ migrating on each substrate (data presented for coating concentrations of 1 and 10 μg/ml). n: number of analyzed cells; N_exp_: number of independent experiments.

REF_WT_ migrated slower, but with higher directional persistence, with increasing FN coating concentration (Fig. 1A,B). A large fraction of REF_WT_ on 10 μg/ml FN polarized towards a random direction, extended large protrusions that appeared to probe the substrate, and often retained their original direction during the 16-hour observation period (Video 1). Interestingly, the increase in directional index (DI: the ratio of start-to-end distance to the total trajectory length) was recorded at FN surface densities, well above those permitting cell adhesion and polarization, and plateaued at densities corresponding to formation of FN monolayers. It is likely that the concentration range examined was not large enough to capture the well-accepted, biphasic response of cell speed, with cells moving faster at optimal, intermediate ligand densities^24^,^25^. On VN-coated substrates, REF_WT_ speed and directional persistence were independent of coating concentration (Fig. 1A,B); fibroblast motion on 10 μg/ml VN was erratic, with cells often changing direction (Video 1). Based on the above, we decided to compare REF_WT_ migration on FN and VN at 10 μg/ml coating concentration, on which the DI on FN was 3-fold higher compared to VN, and in the range where the DI did not increase for FN (Fig. 1A).

Cells were considered motile if they exhibited a maximum displacement of >50 μm (typical cell radius) from the point of origin during the observation period. REF_WT_ motility was stimulated by soluble factors in serum: in absence of serum, or in its presence at 1%, cell speed was dramatically decreased and the majority of cells were immotile (Fig. 1C). In presence of 10% serum, the percentage of motile cells on VN (66%; 53/80) was lower compared to that on FN (87%; 123/140), despite the higher recorded cell speed on VN. This contradiction is due to the analysis method: high speed can result even when cells (nuclei) are wobbling around fixed positions, as often observed on VN. Notably, cell speed and DI were not correlated within given experimental conditions as shown for the cases of FN 1 μg/ml and 10 μg/ml coating concentrations (Fig. 1D).

NIH 3T3 fibroblasts and primary human dermal fibroblasts also exhibited higher directional persistence on FN compared to VN, demonstrating the generality of the effect of coating on directional migration (Supplementary Fig. S2). Overall, our data indicate that directional persistence in fibroblast migration in the absence of soluble or insoluble gradients is enhanced on FN versus VN, and depends on FN surface density.

### Fibronectin promotes formation of polarized protrusions

**Figure 2:**
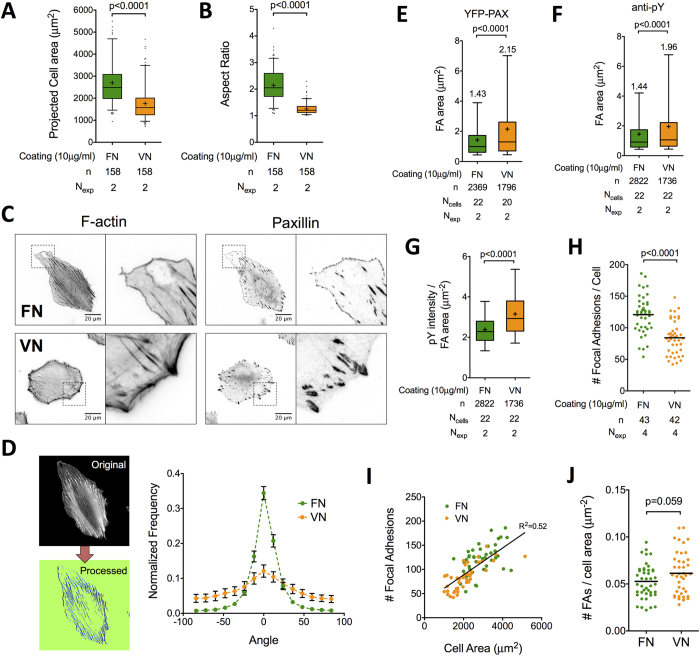
Distinct fibroblast spreading, adhesion plaque organization and cytoskeleton organization on FN versus VN. REF_WT_ projected cell area (**A**) and aspect ratio (**B**) 6 hours post-seeding were significantly higher on FN- compared to VN-coated substrates (n: number of analyzed cells). (**C**) Actin microfilament staining and YFP-PAX localization in REF_YFP-PAX_ 6 hours post-seeding revealed important differences in stress fiber and adhesion plaque organization (see main text for details). (**D**) Stress fiber orientation was quantified using a custom-written algorithm and showed a high degree of fiber alignment on FN and random orientation on VN (details in the materials & methods section; the 0° angle corresponds to the maximum for each cell; mean and SEM from n > 15 cells and 2 independent experiments are presented). (**E**,**F**) FA area quantification based on YFP-paxillin clustering (**E**) or anti-pY staining (**F**) revealed formation of larger FAs on VN compared to FN (n: number of FAs; mean values are shown on graph). (**G**) Quantification of anti-pY fluorescence intensity of FAs normalized to FA area was higher on VN (n: number of FAs from N_cells_). (**H**) Quantification of FA number per cell showed a higher number of FAs present on fibroblasts adhered on FN compared to VN (n: number of cells). However, as the number of FAs per cell was correlated with cell area (**I**), the difference of FA number per unit cell area was not significant between coatings (**J**). The middle line in box plots indicates the median, the box indicates the interquartile range, the whiskers the 5^th^ and 95^th^ percentiles and the cross the mean. Black lines in dot plots (**H,J**) represent mean values. N_exp_: number of independent experiments. Experimental data were analyzed using unpaired t-tests.

In order to help us understand the differences in migratory behavior between coatings, we characterized REF_WT_ adhesion 6 hours after seeding, a time point that allows cell spreading and polarization^26^, but minimizes migration-dependent substrate remodeling (Supplementary Fig. S1). Indeed, fibroblasts remodeled FN coatings and deposited cell-excreted FN as they started to move over the substrate; in contrast, we did not observe remodeling of VN coatings or assembly of FN fibers on VN (Supplementary Fig. S1). REF_WT_ spread more and exhibited higher aspect ratio on FN compared to VN (Fig. 2A,B). REF_WT_ and REF stably transfected with paxillin fused to yellow fluorescent protein (REF_YFP-PAX_) organized filamentous actin into ventral stress fibers oriented along the major cell axis on FN (Fig. 2C & Supplementary Fig. S3). In contrast, ventral stress fibers on VN were scarce and instead, dorsal stress fibers and peripheral bundles were prominent (Fig. 2C & Supplementary Fig. S3). We quantified individual stress fiber orientation^27^ to confirm their higher alignment on FN compared to VN (Fig. 2D). Polarization of fibroblasts and their actin cytoskeleton are consistent with the higher directional persistence measured on FN.

Peripheral focal adhesions (FAs) associated with stress fibers were larger on VN compared to FN (Fig. 2C,E,F). Interestingly, normalized pY intensity per FA area was significantly higher on VN, indicating higher levels of tyrosine phosphorylation and foreseeable differences in signaling (Fig. 2G). The number of FAs/cell was higher on FN (Fig. 2H); however, the linear relationship between FAs/cell and cell area (Fig. 2I) resulted in non-significant differences of FAs/(unit cell area) between the two coatings (Fig. 2J).

Besides FAs, REF_WT_ on FN exhibited multiple, small (<0.4 μm^2^) dot-like adhesions at the edge of large protruding regions free of stress fibers (Fig. 2C & Supplementary Fig. S3). These adhesions persisted in presence of Y-27632, a Rho-kinase inhibitor acting upstream of myosin-II activity, and blebbistatin, a myosin-II inhibitor, suggesting they are nascent adhesions (NAs), which do not require actomyosin contractility for assembly^28^ (Supplementary Fig. S3). Approximately 60% of cells on FN exhibited the characteristic large protrusions with NAs formed at their edge, referred onwards as *polarized protrusions* (Table 1); in contrast, polarized protrusions were absent in cells on VN (Table 1). Polarized protrusion formation required the presence of soluble factors in serum (Supplementary Fig. S3, Table 1). The length of polarized protrusions varied considerably among cells; the distance between the cell edge and the closest elongated FA (as shown in Supplementary Fig. S3) gave an average value of 21 μm for cells on FN (10 μg/ml). Interestingly, on substrates coated with 1 μg/ml FN, half of the cells exhibited polarized protrusions (Table 1), even though they moved randomly with low DI (Fig. 1A).

Overall, fibroblast polarization, cytoskeleton alignment and formation of polarized protrusions on FN versus VN are consistent with the higher directional persistence observed on FN.

### Fibroblast adhesion on fibronectin promotes FAK and paxillin activation

**Figure 3:**
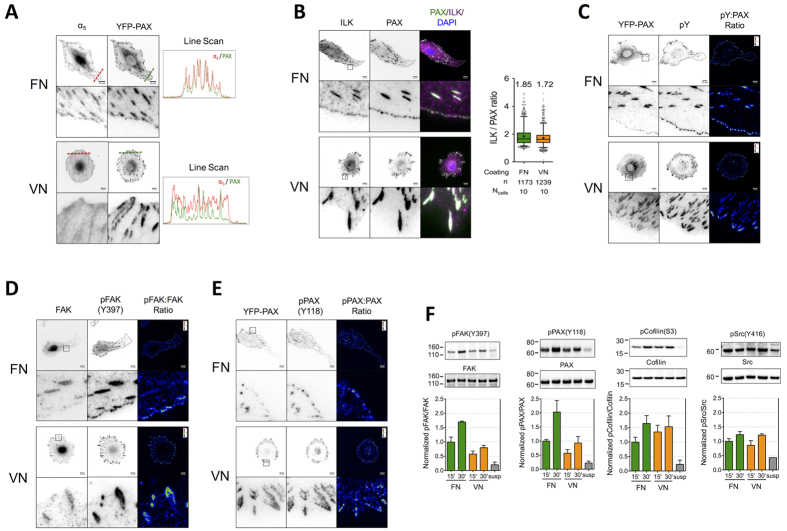
Immunofluorescence microscopy and western blotting of FA components reveal distinct adhesion cluster composition and organization on FN versus VN. REF_YFP-PAX_ (**A,C,E**) or REF_WT_ (**B,D**) were cultured for 6 hours on FN- or VN-coated glass, fixed, stained against indicated FA proteins and examined with epifluorescence microscopy. (**A**) Alpha 5 integrin clustered efficiently on FN but not VN. Normalized intensity profiles along the lines in the images are presented. (**B**) Immunofluorescence imaging of ILK revealed its efficient recruitment to NAs on FN and FAs on both coatings. Quantification of the ILK:paxillin ratio revealed only a minor (8%) reduction in ILK:paxillin ratio per focal adhesion on VN (n: number of analyzed FAs; N_cells_: number of analyzed cells; mean values are indicated on graph), indicating similar recruitment on both coatings. The middle line in box plots indicates the median, the box indicates the interquartile range, the whiskers the 5^th^ and 95^th^ percentiles and the cross the mean. (**C**) Staining against pY and ratio imaging in respect to paxillin revealed enhancement of tyrosine phosphorylation on peripheral NAs compared to mature FAs on FN, and inhomogeneous pY levels within FAs on VN, with the distal part exhibiting higher fluorescence intensity. (**D,E**) pFAK(Y397) and pPAX(Y118) displayed similar distribution as pY. (**F**) Western blot analysis for pFAK(Y397), FAK, pPAX(Y118), PAX, pCofilin(S3), cofilin, pSrc(Y416) and Src from lysates of REF_WT_ in suspension or after plating on FN or VN, for 15 or 30 minutes. Blots are representative of 3 independent experiments and graphs represent their quantification (mean ± SEM). Scale Bars: 10 μm.

In order to identify differences in adhesion signaling between FN and VN, we examined the composition of adhesion plaques through immunofluorescence microscopy and the phosphorylation of key signaling proteins by western blot analysis. Integrin α_5_, which pairs exclusively with β_1_, clustered in adhesions on FN but not VN as expected (Fig. 3A). Moreover, transient expression of green fluorescent protein (GFP)-tagged α_5_ in REF_WT_ showed α_5_ clustering only on FN-coated substrates (Video 2), confirming the specificity of α_5_β_1_ for FN. Α number of antibodies tested for staining against β_3_ or α_v_ integrins (Supplementary information) were not applicable for immunofluorescence of REF_WT_; nevertheless, expression of yellow fluorescent protein (YFP)-tagged β_3_ subunit confirmed efficient α_v_β_3_ clustering on both FN and VN (Video 3). Integrin α_v_β_3_ was additionally present on NAs at the leading edge of transfected cells on FN.

Vinculin staining was faint on NAs compared to FAs (Supplementary Fig. S3), in agreement with reports demonstrating its early recruitment during adhesion formation^29^,^30^, but robust accumulation only upon application of tension to adhesions^31^,^32^. Zyxin, which is also recruited in adhesions under tension^33^, was present in FAs, but not NAs, as expected (Supplementary Fig. S3).

Integrin linked kinase (ILK) was previously shown to preferentially target FAs in cells expressing β_1_-only compared to α_v_-only integrins^13^ and mediate β_1_ but not β_3_ integrin phosphorylation^34^, even though it binds the tail of both integrin types^35^. We therefore considered the possibility that distinct ILK recruitment to adhesions is linked to the observed differences in directional migration between coatings. However, staining against ILK revealed similar recruitment to adhesion clusters on both FN and VN (Fig. 3B), arguing against such a correlation.

NAs on FN stained positive for phosphotyrosine (pY), demonstrating their involvement in adhesion-mediated signaling (Fig. 3C). NAs exhibited higher pY:paxillin intensity ratios compared to FAs, while the distal part of FAs on VN appeared enriched in tyrosine phosphorylated proteins compared to the proximal part (Fig. 3C). Such high pY levels in NAs^36^ and pY polarization within FAs^37^ were previously shown to be necessary, but not sufficient for cell protrusion. We focused on paxillin and focal adhesion kinase (FAK) as two major tyrosine phosphorylated FA proteins that are linked to cell migration. Paxillin is necessary for directional migration^38^ and its phosphorylation at Y118 enhances motility^37^,^39^. FAK activation, indicated by its (auto)phosphorylation at Y398, enhances paxillin phosphorylation^31^ and is also correlated with high directional persistence^40^. Interestingly, FAK activation preferentially occurs following β_1_ and not β_3_ integrin engagement^15^,^41^,^42^, providing a potential mechanism for the observed differences in migration between FN and VN. Immunofluorescence microscopy of REF_WT_ revealed pPAX(Y118) and pFAK(Y397) staining patterns similar to those of pY for FN and VN (Fig. 3D,E), i.e. higher FAK and paxillin phosphorylation on NAs compared to FAs on FN. FAK localization at NAs is in agreement with several other studies^43^,^44^,^45^, but not one study showing FAK recruitment upon tension-mediated maturation^31^.

Western blot analysis revealed increased pPAX(Y118)/PAX and pFAK(Y397)/FAK ratios for REF_WT_ allowed to spread for 30 minutes on FN compared to VN (Fig. 3F). This early time point corresponds to formation of new adhesions to the substrate rather than sustained signaling from FAs. Therefore, our combined results correlate the higher FAK and paxillin phosphorylation, downstream of FN engagement at early adhesions, with the high directional persistence on this coating. FAK and paxillin phosphorylation levels were similar in REF_WT_ cultured 6 hours on FN and VN (Supplementary Fig. S4), in accordance with the decline in FAK activation following initial cell spreading^46^. We hypothesized that FAK activation is necessary for promoting directional migration and examined fibroblast migration in presence of the small molecule FAK inhibitor PF-573228^47^. However, FAK inhibition hindered overall motility, most likely due to reduced FA turnover^48^,^49^ (Supplementary Fig. S4), and therefore it was not possible to link FAK inhibition with directional persistence of fibroblast migration using this approach.

The kinase Src has been shown to cluster on adhesions on VN but not FN^50^, functionally interact with α_v_β_3_ but not α_5_β_1_ integrins and directly bind β_3_ tails^51^. Even though Src often functions in a complex with FAK, it is also able to act independently^31^. Importantly, high Src activity has been linked to loss of directional migration^52^ and therefore we examined whether Src phosphorylation/activation was coating-dependent. Western blot analysis revealed similar pSrc(Y416) levels on FN and VN, indicating that differential Src activation downstream of receptor binding is not linked to the differences in directional migration between the two coatings (Fig. 3F).

Finally, we examined cofilin phosphorylation at S3, which stabilizes actin filaments and was previously suggested to increase following β_1_ but not β_3_ engagement^12^. We observed a strong increase in pcofilin(S3) following REF_WT_ plating, slightly higher levels on VN 15 minutes after plating, but no difference between the two coatings after 30 minutes (Fig. 3F).

Overall, our data demonstrate that distinct receptor engagement on FN versus VN differentially regulates the localization and activation of FAK and paxillin, raising the possibility that the localized activation of these signaling proteins in polarized protrusions is critical to maintain directional migration.

### Focal adhesions turnover more rapidly on FN

Regulation of FA turnover to enable concurrent adhesion assembly at the cell front and disassembly at the rear is necessary for directional migration^1^,^2^,^53^. We evaluated FA stability by quantifying the assembly rate, disassembly rate and lifetime of FAs during initial spreading, as cells spread over pristine protein coating, in order to avoid effects of cell-mediated substrate remodeling and distinctions over leading versus trailing edge FAs. Indeed, we often observed cells moving over, and assembling FAs at regions previously occupied by cells on VN and noticed a clear distinction between leading and trailing edge adhesions on FN (Video 4).

**Figure 4:**
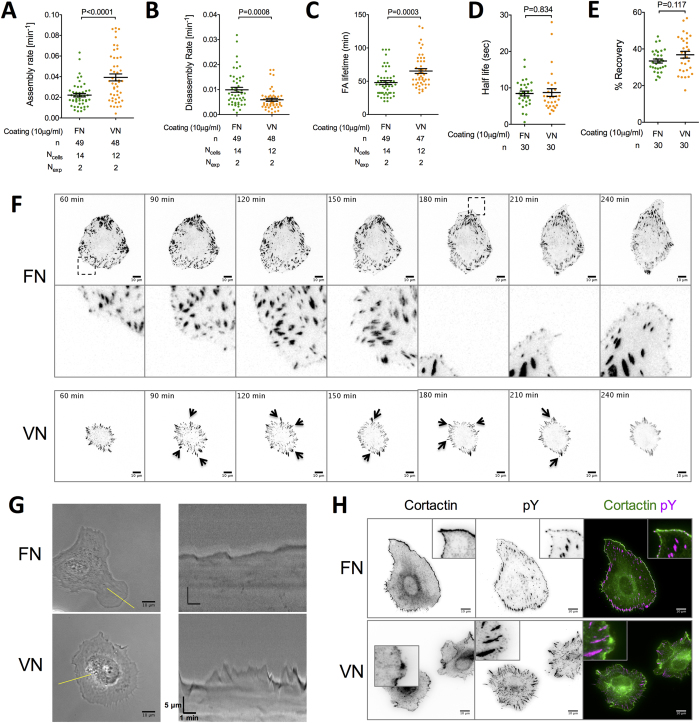
NA localization, lamellipodia dynamics and FA stability depend on the type of adhesive coating. (**A**) FA assembly rates, (**B**) disassembly rates and (**C**) lifetimes were calculated for REF_YFP-PAX_ spreading on FN- and VN-coated glass substrates. (**D,E**) FRAP experiments on mature FAs (>2 μm^2^) close to the periphery of REF_YFP-PAX_ cultured on FN or VN were performed to estimate paxillin turnover (1 of 3 independent experiments presented). Half-life of fluorescence recovery after paxillin photobleaching (**D**) and mobile fraction (**E**) did not show significant differences in paxillin turnover within FAs (n = 30 FAs from 30 cells). (**F**) Individual frames from time-lapse TIRF imaging of REF_YFP-PAX_ on FN- or VN-coated glass substrates. The time after cell seeding is indicated. NAs on FN assembled persistently at the protruding edge (which changed location between 150 and 180 minutes), whereas new adhesions formed randomly around the cell periphery on VN (indicated by arrows). (**G**) Kymograph analysis of REF_WT_ seeded on FN or VN (yellow lines in phase contrast images) revealed smoother lamellipodia and slower protrusion/retraction cycles on FN. (**H**) Immunofluorescence microscopy against cortactin and pY of REF_WT_ cells cultured for 6 hours on FN or VN. Scale bars: 10 μm. Mean and SEM values are presented in dot plots. Experimental data were compared using the unpaired t-test.

FAs assembled faster, persisted longer and disassembled slower on VN (Fig. 4A–C), indicating their higher stability compared to FN. On the other hand, fluorescence recovery after photobleaching (FRAP) analysis revealed no significant differences in the kinetics of paxillin fluorescence recovery, or paxillin mobile fraction between FAs on FN versus VN (Fig. 4D,E & Supplementary Fig. S5). These data suggest that while intracellular accessibility of the adhesome protein paxillin in FAs is similar between coatings, the differential linkage to the ECM promotes faster FA turnover on FN, due to reduced lifetimes and accelerated disassembly.

### Confined NA formation at polarized protrusions stabilizes lamellipodia and determines migration direction

We next asked whether the observed pattern of NAs and polarized protrusions on FN was responsible for maintaining directional persistence of the leading edge. Time-lapse, total internal reflection fluorescence (TIRF) microscopy of REF_YFP-PAX_ revealed that, following initial isotropic spreading, NAs formed and persisted at polarized protrusions on FN, but never on VN (Fig. 4F). Interestingly, the necklace-like pattern of NAs on FN occurred at regions with edge advancement and correlated with the direction of migration, at least for short times (Videos 4,5). As the cell edge moved forward, a fraction of NAs matured to FAs, as previously reported^28^,^33^. On VN, new adhesions formed randomly around the cell periphery and their location was not correlated with whole cell translocation (Video 5).

We reasoned that NAs could promote directional persistence in migration on FN by stabilizing lamellipodia at the leading edge^54^. Indeed, time-lapse, phase contrast microscopy revealed lamellipodia formation limited at protrusive regions on FN, in contrast to the presence of lamellipodia around the perimeter of REF_WT_ on VN (Videos 6,7). Moreover, the frequency of cell edge protrusion/retraction cycles was higher for fibroblasts on VN, and formation of membrane ruffles, which moved centripetally for larger distances, was markedly enhanced on VN compared to FN (Fig. 4G). Accordingly, cortactin, a bona fide lamellipodium marker^55^, localized in patches along the cell perimeter and in structures that resembled membrane ruffles in regions without adhesions on VN (Fig. 4H). In contrast, cortactin was present as a smooth band a few microns in width, at the edges of REF_WT_ on FN-coated substrates, where it co-localized with NAs (Fig. 4H). Notably, cortactin did not require actomyosin contractility or serum for cell edge recruitment on FN (Supplementary Fig. S3).

Overall, the above observations suggest that confined formation of NAs at polarized protrusions contributes to lamellipodia stabilization and correlates with persistent leading edge forward motion on FN, while the absence of these structures on VN promotes randomly oriented adhesion assembly, formation of unstable protrusions and consequently random migration.

### Myosin-II inhibition increases cell speed but impairs directionality

**Figure 5:**
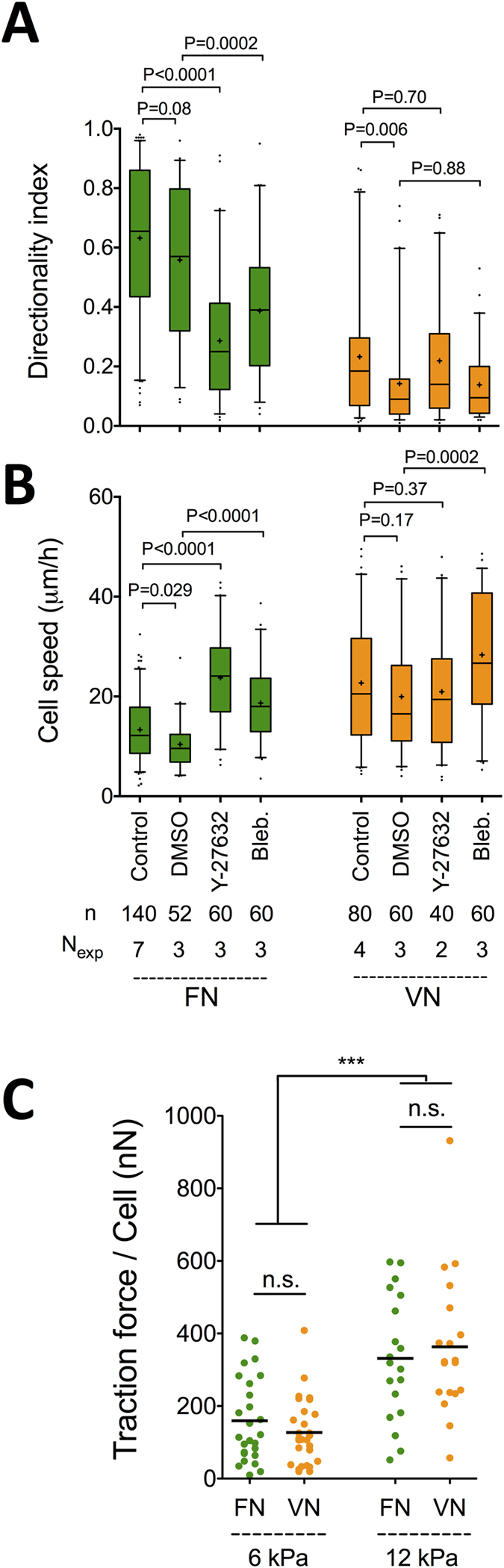
Myosin-II activity is required for directional persistence but cell-level traction forces do not differ between FN and VN. (**A**) REF_WT_ DI and (**B**) cell speed on FN and VN (10 μg/ml) in presence of 0.1% DMSO, 5 μM Y-27632 or 25 μM blebbistatin are presented as box plots (middle line indicates the median, the cross the mean, the box the interquartile range, the whiskers the 5^th^ and 95^th^ percentiles). Data for control conditions are included and are the same as in Fig. 1. Selected columns were compared using unpaired t-tests. n: number of analyzed cells; N_exp_: number of independent experiments. (**C**) Total traction force per cell calculated using traction force microscopy on FN- or VN-coated polyacrylamide substrates of two different elasticities (Young’s moduli of 6 or 12 kPa). An increase in traction force for the stiffer gels was observed but no significant differences between coatings. Experimental data on different coatings were compared using unpaired t-tests and between different elasticities using one-way ANOVA with Tukey post-test analysis (n.s.: not significant; ***p < 0.001).

We considered the possibility that myosin-II inhibition enhances directional migration by promoting NA assembly rather than FA maturation. On the other hand, myosin-II activity is necessary for polarity establishment^37^,^56^, and directional persistence in cell migration requires elevated substrate stiffness, and hence cell contractility^57^,^58^,^59^,^60^. Myosin-II inhibition using blebbistatin or Y-27632 resulted in a pronounced decrease of directional persistence and an increase of REF_WT_ speed on FN (Fig. 5A,B), indicating the requirement of myosin-II activity for directional migration. The inhibitors did not result in significant changes in directional persistence on VN (Fig. 5A). Cell speed was significantly increased for blebbistatin-treated cells but not Y-27632-treated ones on VN, suggesting possible inhibitor-specific differences (Fig. 5B). Interestingly, addition of 0.1% DMSO (dimethyl sulfoxide), used as a vehicle for blebbistatin, reduced the percentage of REF_WT_ that exhibit polarized protrusions on FN to 36% (Table 1, Supplementary Fig. S7). Nevertheless, fibroblasts exhibited high DI values, suggesting that polarized protrusions are not necessary for high directional persistence. At higher DMSO concentrations, a negative effect on directional migration was recorded, highlighting that care should be taken when using DMSO in cell migration studies (Supplementary information).

Next, we tested whether coating-dependent regulation of myosin-II-mediated traction forces dictates the biochemical events that maintain directionality^61^. Activation of β_1_–but not β_3_–integrins was shown to enhance traction forces in a FN concentration-dependent manner^62^ and integrin α_5_β_1_ proved to be a stronger puller compared to α_v_β_3_^63^. These findings are consistent with a scenario where α_5_β_1_ engagement on FN allows for higher traction forces and subsequent directional persistence compared to VN. However, we observed similar levels of cellular traction forces on FN- and VN-coated polyacrylamide hydrogels, using traction force microscopy (TFM) (Fig. 5C, Supplementary Fig. S6). Force generation was higher on stiffer substrates as expected^13^,^64^ (Fig. 5C).

Overall, our data indicate that while actomyosin contractility is required for high directional persistence in fibroblast migration on FN, differences in the magnitude of applied traction forces is not responsible for coating-specific differences in directional migration.

### Blocking α_v_β_3_ or α_5_β_1_ integrins reduces directional persistence on FN

Up to here our experiments have not addressed which specific receptors are responsible for the observed coating-dependent differences in fibroblast adhesion and migration, and in particular the high directional persistence observed on FN. Besides α_5_ integrin (Fig. 3), we confirmed that REF_WT_ express β_1_, β_3_ and α_v_ integrins by western blot analysis (Supplementary Fig. S8). Several receptors can recognize FN, including various integrins and syndecan-4; we here focused on the effects of α_v_β_3_ and α_5_β_1_ integrins, due to their proposed role in directional migration^11^,^12^ and the availability of highly selective, small-molecule, integrin antagonists against these integrins^14^,^65^. These antagonists were originally designed to have high affinity and selectivity against the RGD-binding site of α_v_β_3_ or α_5_β_1_ integrin, with low α_IIβ_β_3_ affinity^66^, and possess low activity towards other RGD-binding integrins (data not shown). REF_WT_ adhesion to FN was drastically inhibited upon incubation with the α_5_β_1_ but not the α_v_β_3_ selective antagonist, suggesting that α_5_β_1_ is the major FN-binding integrin (Supplementary Fig. S8).

**Figure 6:**
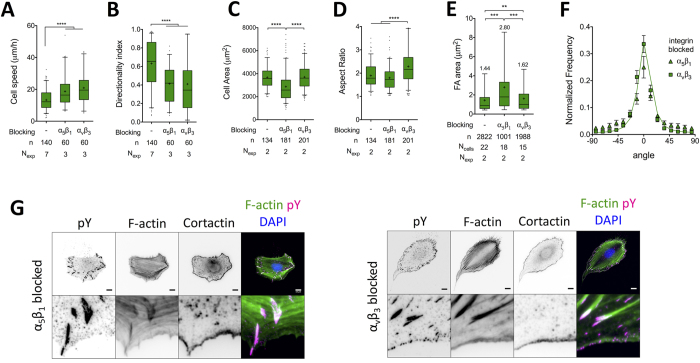
Blocking of α_5_β_1_ or α_v_β_3_ integrins inhibits directional migration. (**A**) REF_WT_ cell speed and (**B**) DI on FN (10 μg/ml) in presence of indicated soluble, integrin-selective antagonists. A significant increase in cell speed and a decline in directional persistence was observed following α_5_β_1_ or α_v_β_3_ blocking. Data for control (FN) are included for comparisons and are the same as in Fig. 1. (**C**) Projected cell area and (**D**) aspect ratio of REF_WT_ cultured for 5 hours on FN and then incubated for 1 hour with soluble integrin antagonists. Blocking of α_5_β_1_ resulted in a substantial cell area reduction, while blocking of α_v_β_3_ in an increase of aspect ratio. (**E**) Quantification of FA area based on anti-pY staining revealed a pronounced increase in FA size following α_5_β_1_ blocking (n: number of analyzed FAs; mean value indicated on graphs). (**F**) Stress fiber orientation was not affected by α_v_β_3_ blocking, but became more random following α_5_β_1_ blocking. Mean and SEM values from at least 10 cells from 2 independent experiments are presented; the green line represents data for the FN control (same as in Fig. 2D). (**G**) Representative immunofluorescence images of REF_WT_ after selective integrin blocking show adhesion plaque and actin cytoskeleton remodeling following α_5_β_1_ but not α_v_β_3_ blocking. The middle line in box plots indicates the median, the box indicates the interquartile range, the whiskers the 5^th^ and 95^th^ percentiles and the cross the mean. N_exp_: number of independent experiments. Experimental data were compared using one-way ANOVA with Tukey’s post-test analysis (**A–D**) or with Bonferroni’s post-test analysis (**E**). Only statistically significant differences are shown: **P < 0.01, ***P < 0.001, ****P < 0.0001. Scale bars: 10 μm.

Blocking of α_v_β_3_ integrins on spread fibroblasts caused a decrease in directional persistence and a concomitant increase in cell speed (Fig. 6A,B), consistent with its role in promoting directional migration^12^ and a previous study showing impaired directional persistence of NIH3T3 cells following peptide-mediated α_v_β_3_ blocking^67^. Interestingly, blocking α_v_β_3_ did not considerably affect cell size (Fig. 6C,G), FA size (Fig. 6E), or stress fiber orientation (Fig. 6F), while a slight increase in both the aspect ratio (Fig. 6D) and the formation of polarized protrusions was recorded (Table 1). These findings are consistent with the proposed mechanism for α_v_β_3_-mediated loss of directional persistence involving altered α_5_β_1_ trafficking rather than remodeling of adhesion structures and actin cytoskeleton^67^.

We reasoned that blocking α_5_β_1_ integrins would favor cell binding to FN through α_v_β_3_ integrins^68^, and consequently directional persistence would be high^12^,^13^. However, α_5_β_1_ blocking caused significant loss of directional persistence and an increase in cell speed (Fig. 6A,B), indicating that substrate engagement of α_5_β_1_ integrins is necessary for high directional persistence in migration. While the DI was comparable following α_v_β_3_ or α_5_β_1_ blocking, the effect of the two antagonists on cell morphology, actin cytoskeleton and adhesion plaque organization were dissimilar. Blocking of α_5_β_1_ resulted in a large reduction of REF_WT_ area (Fig. 6C) and the elimination of NAs (Fig. 6G) and polarized protrusions (Table 1), indicating that α_5_β_1_ is required for their assembly. FAs increased dramatically in size, suggesting that absence of α_5_β_1_ engagement stabilizes FAs on FN (Fig. 6E,G). Finally, stress fiber orientation became more random, often resembling cells seeded on VN (Fig. 6F,G). The above results indicate that α_5_β_1_ blocking causes remodeling of adhesion structures and actin cytoskeleton, which lead to a decrease in directional persistence. Notably, these effects were observed following 1-hour incubation with the integrin antagonists on spread fibroblasts, and therefore highlight FA remodeling events rather than altered assembly.

The above data demonstrate that engagement of FN by both α_5_β_1_ and α_v_β_3_ integrins is required for high directional persistence in fibroblast migration, but the mechanism of action for each integrin appears to be distinct.

### Fibroblast adhesion and migration on immobilized α_5_β_1_ and α_v_β_3_ selective ligands

**Figure 7:**
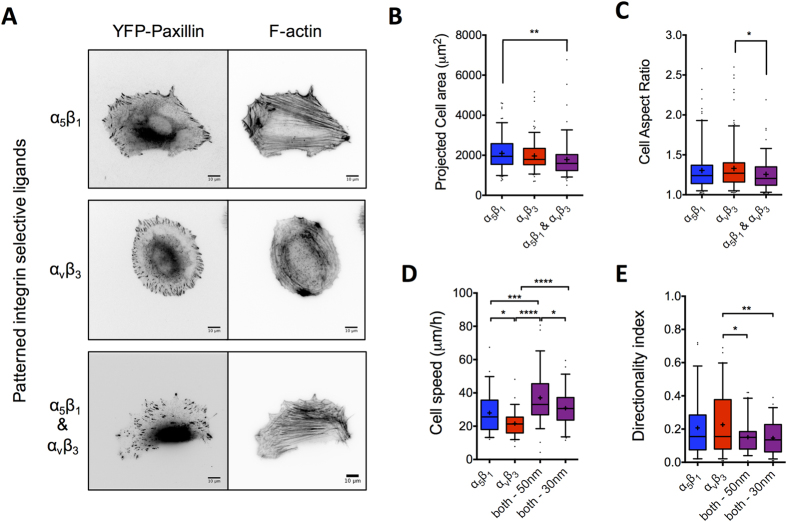
Immobilized α_5_β_1_ and α_v_β_3_ integrin selective ligands on patterned substrates do not promote high directional persistence in fibroblast migration. (**A**) REF_YFP-PAX_ actin cytoskeleton and YFP-paxillin clustering 6 hours post-seeding on patterned gold particles with a 50 nm average inter-particle distance, and functionalized with the indicated ligands. Stress fiber morphology on α_5_β_1_- and α_v_β_3_-selective surfaces resembles that on FN and VN, respectively. FAs, but not NAs nor polarized protrusions, are present on patterned substrates, independent of the type of immobilized ligands. (**B,C**) Quantification of REF_WT_ projected cell area (**B**) and aspect ratio (**C**) 6 hours post-seeding on patterned gold particles with a 50 nm average inter-particle distance (n > 100 cells). (**D**) Cell speed increased on patterned substrates with a 50 nm inter-particle distance presenting both α_5_β_1_- and α_v_β_3_-selective ligands (1:1 ratio) compared to substrates presenting these ligands alone (n = 60 cells from N_exp_ = 3). Decreasing the inter-particle distance to 30 nm resulted in a slight decrease in cell speed when both ligands were present (n = 60 cells from N_exp_ = 2). (**E**) DI remained very low for REF_WT_ on all substrates, independent of integrin ligand type or density, indicating random migration on patterned substrates. The middle line in box plots indicates the median, the box indicates the interquartile range, the whiskers the 5^th^ and 95^th^ percentiles and the cross the mean. Experimental data were compared using one-way ANOVA with Tukey’s post-test analysis. Only statistically significant differences are shown: *P < 0.05; **P < 0.01, ***P < 0.001, ****P < 0.0001. Scale bars: 10 μm.

We next examined whether substrate engagement of α_5_β_1_ and α_v_β_3_ integrins is sufficient to promote directional migration. REF_WT_ were presented with immobilized α_v_β_3_ and/or α_5_β_1_ integrin-selective ligands on gold nanoparticles, which were hexagonally-patterned on a substrate passivated with poly(ethylene glycol) (PEG)^65^,^69^. REF_WT_ spread efficiently on patterned substrates with an inter-particle spacing of 50 nm (Fig. 7A,B). REF_WT_ area was similar between α_5_β_1_ and α_v_β_3_ patterned substrates, and slightly decreased on substrates functionalized with a 1:1 mixture of the two ligands (Fig. 7B). Fibroblasts remained rounded, with much lower aspect ratios compared to fibroblasts spread on FN (Fig. 7C). The actin cytoskeleton of fibroblasts on α_5_β_1_- and α_v_β_3_-selective ligands resembled those plated on FN and VN, respectively, indicating that α_5_β_1_ engagement is sufficient to promote formation of aligned ventral stress fibers (Fig. 7A). However, we did not observe formation of NAs or polarized protrusions on any of the patterned substrates independent of functionalized ligand (Table 1). Single cell migration assays revealed a small difference between the α_5_β_1_- and α_v_β_3_-selective substrates in respect to speed and no change in directional persistence, with DI values being very low in comparison to FN-coated TCPS (Fig. 7D,E). Pattern functionalization with both ligands resulted in higher cell speed and a lower DI (Fig. 7D,E). We tested REF_WT_ migration on patterned substrates exhibiting two different average inter-particle distances (30 and 50 nm) in order to ensure that the low DI is not due to the presented ligand density (Fig. 7E). REF_WT_ migrated slower on the more densely functionalized patterns, most likely due to higher adhesion strength^25^, but did not increase their directional persistence (Fig. 7D). In summary, our results suggest that substrate engagement of integrins α_5_β_1_ and α_v_β_3_ is not sufficient to promote high directional persistence in migration, compared to FN.

## Discussion

In this study we have demonstrated that fibroblasts exhibit high directional persistence in migration on FN- but not VN-coated substrates and that engagement of both α_v_β_3_ and α_5_β_1_ integrins is necessary for directional migration on FN. We avoided exogenous integrin expression, silencing or knockout; instead, fibroblasts were presented with different substrates, unveiling the impact of presented ligands and their corresponding receptors on adhesion and motility regulation. While we identified several important differences in adhesion phenotype, fibroblast morphology and actin cytoskeleton organization as a function of substrate composition and selective integrin engagement, it was not possible to correlate these phenotypes with high directional persistence over longer time scales (hours).

Fibroblasts on FN organized filamentous actin into oriented, ventral stress fibers, in a process that required substrate engagement of α_5_β_1_ integrin (Figs 2 and 6). This is in line with the ability of α_5_β_1_ to selectively activate RhoA^70^ and the role of RhoA in formation of this type of stress fibers^71^. Given the interest in controlling stress fiber morphology^72^,^73^ and the emerging role of stress fibers in mechanosensing^71^, our observations are potentially useful in directing stress fiber assembly through modifying substrate coating and integrin engagement.

FAs were larger and more stable on VN compared to FN, consistent with α_v_β_3_ integrin being the main VN receptor and reports that β_3_ integrins are enriched and more static on FAs compared to β_1_ integrins^74^,^75^,^76^. Accordingly, FAs on FN grew considerably in size upon α_5_β_1_ blocking (Fig. 6). Increased FA stability on VN is expected to inhibit FA re-orientation parallel to the direction of movement, FA turnover and subsequent directional motility, as was indeed observed.

Our study identified high FAK phosphorylation on FN versus VN, as a potential signaling mechanism that promotes directional migration on the former coating^40^, which was also consistent with the higher observed pPAX(Y118) levels and faster FA turnover^39^,^48^. Interestingly, FAK-null keratinocytes exhibited very similar phenotype to fibroblasts cultured on VN presented here, with impaired cytoskeleton organization, FA disassembly and directional migration, reinforcing the idea that differential FAK activation is responsible for migration differences between FN and VN^49^. The mechanistic link between increased FAK activation and directional persistence of fibroblast migration was not established here; candidate signaling pathways linked to cell migration, downstream of FAK activation include FAK-mediated regulation of the Arp2/3 complex^77^, cortactin^78^, the FAK-p130Cas-Rac-Lamellipodin signaling module^79^ and RhoA^80^.

A compelling feature of fibroblasts cultured on FN was the presence of polarized protrusions with NAs at their edge. Their assembly required α_5_β_1_ integrin binding, in line with Rac activation downstream of β_1_ integrin engagement^13^,^81^,^82^,^83^ and subsequent Rac-mediated NA and lamellipodia assembly^84^. Engagement of α_5_β_1_ integrin on patterned susbstrates was, however, not sufficient for NA or polarized protrusion formation (Fig. 7). One possible reason is that the discrete nature of integrin binding sites on patterned substrates impedes formation of NAs. Alternatively, competent Rac activation might require additional signals beyond those downstream of α_5_β_1_ integrin. Interestingly, while NAs formed on FN even in absence of serum, polarized protrusion formation required soluble factors present in serum, further supporting this hypothesis.

Actin polymerization was previously shown to transport β_1_, but not β_3_ integrins at the leading edge for cells to probe permissive adhesion sites^85^, which in our experiments would be present on FN but not VN, allowing for substrate anchoring and NA assembly. We nevertheless observed also α_v_β_3_ in NAs (Video 3), in agreement with its reported localization at the leading edge following Rac activation^86^, and with recently presented evidence that α_5_β_1_ is able to recruit α_v_β_3_ at adhesion sites^14^. Overall, our results are consistent with a model where α_5_β_1_ is transported to the leading edge, engages FN and contributes to Rac activation, NA formation and rapid recruitment of α_v_β_3_. An appealing hypothesis, which could additionally account for the ligand density dependence of directional persistence, is that a certain number of engaged receptors is necessary to activate *optimal* Rac levels and formation of a single leading edge^10^,^87^.

Surprisingly, the presence of polarized protrusions was not sufficient, nor necessary to ensure long-term directional persistence based on the following observations: *i*) fibroblasts treated with α_v_β_3_ integrin antagonists exhibited reduced directional persistence (Fig. 6), even though they readily formed polarized protrusions (Table 1), *ii*) reducing FN coating concentration to 1 μg/ml had a large impact on directional persistence (Fig. 1), but inhibited mildly polarized protrusion formation (Table 1), and *iii*) fibroblasts treated with 0.1% DMSO exhibited impaired formation of polarized protrusions but maintained a polarized morphology and high directionality (Supplementary Fig. S7). Nevertheless, the location of polarized protrusions was predictive of local, persistent edge advancement and short-term, persistent motility along their orientation. A high density of NAs at the cell edge could concentrate the necessary biochemical signals for sustained Rac activation^54^,^88^; Rac activates cortactin that in turn activates the Arp2/3 complex, resulting in persistent lamellipodia at the leading edge^89^,^90^,^91^. Alternatively, but not mutually exclusive, NAs could promote lamellipodia persistence by acting as physical brakes in retrograde actin flow^92^ or actin arc flow^93^. Actin polymerization against the membrane is counteracted by membrane tension, leading to characteristic temporal protrusion-retraction cycles that advance the cell edge through formation of new adhesions^93^,^94^. In the absence of NAs to anchor the membrane, these cycles are deregulated with membrane ruffles pulling back over the cell body and impeding the process of smooth edge advancement, as evidenced for cells cultured on VN.

Our work indicates that directional migration on FN requires the engagement of both α_v_β_3_ and α_5_β_1_ integrins to the substrate, and does not support the *general* view that β_3_ integrins promote directional migration, while β_1_ integrins favor random migration^11^,^12^,^13^. This conclusion is in line with a very recent study reporting β_1_ integrin retrograde transport as essential to maintain persistent cell migration^95^ and previous work demonstrating that cells exhibit low directional persistence on patterned surfaces of VN (α_v_β_3_-binding) or collagen (β_1_-binding) compared to high persistence on surfaces where these proteins were combined^96^. REF_WT_ did not exhibit high directional persistence on VN or α_v_β_3_-selective, patterned substrates, despite α_v_β_3_ integrin surface engagement in both cases. At the same time, α_v_β_3_ binding to FN was necessary for high directional persistence (Fig. 6) as previously shown^67^,^97^,^98^. The reason for the discrepancy between our work and previous studies reporting high directional persistence downstream of β_3_ but not β_1_ integrins remains unclear and could be due to differences in cell type or effects from integrin over-expression. For example, β_1_ integrins increased RhoA activity but maintained low Rac activity in cells of epithelial origin^12^, in contrast to results from experiments following integrin expression in a pan-integrin-null fibroblast background^13^. Alternatively, absence of additional, relevant integrin heterodimers in addition to the studied (expressed) integrin, might influence essential receptor cross-talk and intracellular signaling pathways.

Patterned substrates designed to selectively engage α_v_β_3_ and α_5_β_1_ integrins failed to promote high directional persistence. Even though we cannot exclude that the mode of ligand presentation, or that ligand affinity and density were not optimal, the very low directional persistence observed strongly suggests that integrin engagement alone is not sufficient for promoting directional migration. A possibility is that additional signals from other cell surface receptors are necessary to co-ordinate with those downstream of integrin engagement. A candidate receptor is syndecan-4, which binds the heparin-binding domain of FN, is involved in Rac-1 activation, p190RhoGAP regulation at the leading edge and concomitant directional persistence^99^,^100^,^101^,^102^. Another candidate receptor, which binds the alternatively spliced CS-1 region of FN-and not the RGD sequence-is integrin α_4_β_1_. Localized α_4_ phosphorylation at the leading edge regulates paxillin binding and subsequent lamellipodial persistence through integrin-paxillin-Arf GAP complex regulation of Rac^103^,^104^. However, we note that high directional persistence on FN could also be a result of cell-mediated FN remodeling or FN-mediated growth factor immobilization on its 12^th^–14^th^ type III domain^105^. Our ongoing work is focused on elucidating which regions of FN, and which receptors on the cell surface are responsible for the observed directional migration phenotype.

In summary, our data indicate that dense FN-coated substrates promote high directional migration, in a process that requires the co-operative action of engaged α_5_β_1_ and α_v_β_3_, along with additional FN-binding receptors. We have identified increased FAK and paxillin phosphorylation as a potential substrate-dependent regulator downstream of differential substrate engagement, and showed that myosin-II activity is necessary for maintaining directionality on FN. α_5_β_1_-mediated NA and polarized protrusion formation was linked to edge advancement and short-term directional migration, but was not necessary for long-term directional persistence. In general, our findings highlight that conclusions on cell migration from a specific coating cannot be generalized and suggest that the role of specific integrins depends on the type of adhesive substrate. Evidently, fibroblast migration *in vivo* differs dramatically from the reductionist approach employed here. Nevertheless, our results contribute insight on the mechanisms of cell adhesion and migration as a function of differential ligand engagement and further hint at design criteria when considering functionalization of biomaterials where directional migration is desired.

## Materials and Methods

### Reagents, Antibodies & Plasmids

Fibronectin from bovine plasma (Cat# F1141), DMSO, rhodamine-phalloidin, Y27632 dihydrochloride, PF-573228, bovine serum albumin (BSA), accutase and blebbistatin were purchased from Sigma. Vitronectin from human plasma (Cat# PHE0011), wheat germ agglutinin alexa fluor 488 conjugate (WGA) and (4′,6-diamidino-2-phenylindole) (DAPI) were purchased from Life Technologies. The α_5_-integrin-GFP plasmid (plasmid 15238)^106^ and β_3_-integrin-YFP plasmid (plasmid 26653)^107^ were obtained from Addgene. Integrin α_5_β_1_ and α_v_β_3_ selective ligands were prepared as previously described^65^,^66^, and their structures shown in Supplementary scheme 1.

The following antibodies were used for immunofluorescence (1:100 dilution) and western blotting (1:1000 dilution): monoclonal anti-paxillin [165/Paxillin] (BD Biosciences), polyclonal anti-phospho-paxillin (Y118) (Millipore), monoclonal anti-FAK [77/FAK] (Bd Biosciences; immunofluorescence), polyclonal anti-FAK (Y397) (Cell Signaling; western blotting), polyclonal anti-phospho-FAK (Y397) (Sigma; immunofluorescence), monoclonal anti-Src [L4A1] (Cell signaling), polyclonal anti-phospho-Src (Y416) (Cell Signaling), polyclonal anti-Cofilin (Cell signaling), polyclonal anti-phospho-Cofilin (S3) (Cell Signaling), monoclonal anti-zyxin [164D4] (Synaptic Systems), polyclonal anti-cortactin (Santa Cruz Biotechnology), polyclonal anti-integrin alpha 5 (Chemicon), monoclonal anti-phosphotyrosine [PY99] (Santa Cruz Biotechnology), monoclonal anti-vinculin [hVIN-1] (Sigma), monoclonal anti-ILK [EPR1592] (Millipore), monoclonal anti-fibronectin [P1H11] (Millipore; ELISA), polyclonal anti-bovine fibronectin (Millipore; immunofluorescence), monoclonal anti-cellular fibronectin [DH1] (Millipore; immunofluorescence), and polyclonal anti-vitronectin (Santa Cruz Biotechnology).

### Substrates

FN and VN were coated on: 1) TCPS Petri dishes (Greiner Bio-one), 2) 96-well cell culture plates (Greiner Bio-one), 3) Glass-bottom WillCo dishes or 4) Chambered glass cover slips (Nunc). Substrates were incubated with freshly prepared FN or VN PBS solutions overnight at 4 °C, blocked with 1% BSA for 15 min at 37 °C, washed with PBS and used within 1 day of preparation.

In order to measure relative coating efficiencies, FN- or VN-coated wells, blocked with BSA, were incubated with 0.1 μg/mL of anti-FN or 0.02 μg/ml of anti-VN for 1 hour at room temperature, followed by washing with PBS and incubation with secondary antibodies coupled to horseradish peroxidase (HRP) (0.16 μg/mL for FN-coated and 0.4 μg/ml for VN-coated; Santa Cruz Biotechnology) for 1 hour at room temperature. Wells were washed with PBS and secondary antibodies were detected using a TMB substrate (3,3′,5,5′ -tetramethylbenzidine; Sigma) and absorbance measurements at 630 nm.

Patterned gold nanoparticle substrates were prepared using block copolymer micelle nanolithography (BCML) as previously described^69^. Substrates with inter-particle spacing of 30 nm (33 ± 4 nm; mean ± standard deviation; n = 3) or 50 nm (47 ± 8 nm; mean ± standard deviation; n = 3) and passivated between particles using polyethylene glycol (PEG) were prepared. Solutions (25 μM) of integrin α_5_β_1_ and α_v_β_3_ selective antagonists modified with thiols^65^ were incubated with gold nanoparticles for 4 hours at room temperature to allow coupling, followed by washing with PBS containing 1% BSA. Functionalized substrates were air dried and either glued over a hole in a Petri dish for migration studies or placed in 6-well plates for immunofluorescence studies. Functionalized and passivated patterned substrates were used within 1 day of preparation.

## Cells

The rat fibroblast cell line REF52 (REF_WT_) and REF52 stably transfected with paxillin fused to yellow fluorescent protein (REF_YFP-PAX_) were cultured as sub-confluent monolayers in Dulbecco’s modified eagle’s medium (DMEM; Life Technologies; Prod. #10938), supplemented with 10% fetal bovine serum (FBS) and 1% penicillin/streptomycin (P/S). Cells were serum-starved overnight (12–16 hours) prior to seeding, unless noted otherwise. Cells were detached using accutase treatment and seeded in serum-free DMEM. NIH 3T3 mouse fibroblasts were cultured in DMEM (Life Technologies; Prod. # 41966), supplemented with 10% fetal calf serum (FCS) and 1% P/S. Cells were serum starved 3 hours prior to seeding. Primary human dermal fibroblasts (pHDF) were purchased from ATCC (Cat # PCS-201-010) and cultured according to instructions provided. Fibroblasts basal medium (ATCC Cat # PCS-201-030) was supplemented with fibroblast growth kit-low serum (ATCC Cat # PCS-201-041) and 1% P/S. pHDF cells were used until passage 8. pHDF cells were not serum starved prior or during cell motility experiments. All cell lines were kept at 37 °C and 5% CO_2_, in a humidified atmosphere.

### Transfection

REF_WT_ were cultured in standard 6-well plates for 24 h prior to transfection. Promofectin (PromoKine) was used to transfect cells according to the manufacturers’ protocol. Briefly, plasmids (1:20 in DMEM) were mixed with promofectin (1:10 in DMEM) and incubated for 15 min at room temperature. The transfection complex was then added (1:200 in supplemented DMEM) to REF_WT_ and incubated for 24 hours, prior to cell detachment, seeding and imaging.

### Cell Adhesion Assay

Relative efficiency of REF_WT_ cell adhesion was analyzed by quantifying the number of cells attached on substrates 20 minutes after seeding. Briefly, REF_WT_ were detached and kept in suspension under ice for 10 minutes, with or without integrin selective antagonists. Next, REF_WT_ were incubated with coated wells (96-well culture plate) under serum-free conditions for 20 minutes. Wells were washed twice with ice-cold PBS and dried culture plates were placed at −80 °C overnight. Relative cell numbers were quantified using the Cyquant cell proliferation assay kit (Life Technologies) according to the instructions provided.

### Single Cell Motility Assay

Fibroblasts were plated on substrates in serum-free medium at a density of 1–2 × 10^3^ cells/cm^2^. After 30 minutes, non-adherent cells were removed by aspiration and supplemented medium was added (except for pHDF; see above). Cells were imaged using phase contrast, time-lapse microscopy at 37 °C, in presence of 5% CO_2_. Images were acquired every 10 minutes for 16 hours, starting 4 hours after cell plating. A Delta Vision (DV) system (Applied Precision Inc.) on an Olympus IX inverted microscope equipped with a cooled CCD camera and a 10x/0.3 NA (Olympus) objective were used. Cell trajectories were obtained using the ‘manual tracking’ plugin of ImageJ software and monitoring the displacement of the nucleus in each frame. Cells that 1) remained within the field of view, 2) did not divide and 3) were viable throughout the experiment were analyzed in the case of REF_WT_ and NIH3T3 fibroblasts. pHDF cells divided much more frequently, therefore trajectories of cells were included prior to cell division, provided that the time of observation was >6 hours. Speed was calculated as the total path length divided by time, and directionality index (DI) as the ratio of the distance from the origin to the total trajectory length. For inhibition or blocking studies, the corresponding molecules were added 1 hour before time-lapse imaging.

### Lamellipodia dynamics

Cells seeded for 4–5 hours on FN- or VN-coated glass substrates were imaged at 37 °C, in CO_2_-independent medium, supplemented with 10% FBS using a Zeiss AxioObserver Z1 microscope equipped with a 63×/1.4 NA oil objective. Kymograph analysis was performed using the “multiple kymograph” plugin of ImageJ.

### Immunofluorescence microscopy

Cells cultured on surfaces for 6 hours were washed once with PBS and fixed with 4% paraformaldehyde. Membranes were permeabilized using Triton X-100 (0.1%) followed by BSA blocking (3%). Primary antibodies (in 1% BSA) were incubated for 1–2 hours at room temperature or overnight at 4 °C, and secondary alexa fluor®-labeled antibodies were incubated for 1 hour at room temperature. DAPI and phalloidin were used to stain nuclei and filamentous actin (F-actin), respectively. Images were acquired on the DV system described above, using a 60x/1.4 NA (Olympus) oil-immersion objective.

### Image analysis

Cell projected area and aspect ratios were determined through image analysis of Phalloidin- or WGA-labeled cells using the ‘Cell Outliner’ plugin of ImageJ. Ratio imaging was performed using a custom-written ImageJ plugin. Focal adhesion analysis was performed using a processing script written in Python, using the Python imaging library and numpy. The basic functions are available as part of the ImageP package on Launchpad. Immunofluorescence images were first background corrected using a rolling ball filter (diameter of 32 pixels) and then smoothed using a Gauss kernel with a 5-pixel radius and standard deviation of 1 pixel. Adhesions were identified as bright pixels after applying automatic threshold using Otsu’s method. An area threshold of 0.4 μm^2^ was set to exclude small adhesions and noise. Bright spots were localized and used as binary masks for calculating sum intensities from the original images.

Stress fiber orientation was calculated based on a published method^27^. Briefly, images were background corrected using a rolling ball filter and smoothened with a Gaussian filter (standard deviation of 0.75 pixels). Then an asymmetric Mexican hat filter (a Laplace operator applied to a 2-dimensional Gaussian function) with standard deviations of 10 pixels in x-direction and 0.75 pixels in y-direction, rotated to 15 angle values between −90 and 90 degrees, was convolved to the images. The maximum of each pixel along the various angles were recorded (maximum image), as well as the angle where this maximum was found (angle image). The maximum image was then filtered using an automatic threshold (Otsu’s method), and only values above this threshold were kept. The remaining angle values were converted to a histogram, which was rotated such, that the maximum was directed to 0 degrees.

### TIRF microscopy

Fibroblasts were imaged using the DV system described above in TIRF mode using a blue laser (488 nm) and a 60 ×/1.4 NA oil-immersion objective (Olympus). For FA dynamics analysis, individual FAs that assembled and disassembled as cells spread over the surface were analyzed in respect to fluorescence intensity. The resulting intensity profiles versus time were analyzed to determine assembly rate, disassembly rate and steady state lifetime, by fitting the corresponding regions with linear fits.

### FRAP measurements

FRAP measurements were performed on live REF_YFP-PAX_ plated for 4–6 hours on coated glass substrates. Imaging was performed using the DV system and a 60×/1.4 NA oil-immersion objective (Olympus). A spot of approximately 1 μm in diameter on mature FAs was bleached using a short laser pulse (488 nm; 50 ms). Images were acquired every 100 ms just before and immediately after the bleaching event to monitor fluorescence recovery. Fluorescence intensity of both the bleached FA and a neighboring FA were recorded to correct for overall fluorescence intensity loss during acquisition. FRAP curves of individual FAs were normalized to their intensity before the bleach event and experimental data were fit using the equation y = y_0_ + (y_p_ − y_0_)*(1-exp(−kt)), where y_0_ the intensity of the FA immediately after photo-bleaching, y_p_ the plateau intensity and (y_p_ − y_0_) the mobile fraction. The recovery half-time is calculated as ln(2)/k.

### Western Blotting

REF_WT_ were serum-starved overnight. Cells were detached from the substrate using trypsin, which was neutralized with serum-supplemented medium. Following centrifugation and resuspension in serum-supplemented medium, cells were seeded on FN- or VN-coated Petri dishes (1–2 million cells per dish of 60 mm in diameter) and incubated at 37 °C and 5% CO_2_ for 15 or 30 minutes. Cells were also kept in suspension in ice for 15 minutes to serve as a control. Adherent cells were washed three times with ice-cold PBS, followed by incubation for 30 minutes with the lysis buffer (1% IGEPAL CA-630, 0.25% sodium deoxycholate, 667 mM EDTA, 100 mM PMSF, 200 mM Na_3_VO_4_, 150 mM NaCl; all reagents from Sigma) and scraping from the surface. Suspension cells were centrifuged (1200 rpm) and resuspended in PBS twice prior to addition of the lysis buffer. Lysates were centrifuged (5000 rcm for 15 s), the supernatant collected and its protein concentration determined with the BCA assay (Thermo Scientific). Proteins were loaded onto polyacrylamide gels for electrophoresis (NuPAGE 4–12% Bis–Tris Gel; Life Technologies) and then transferred to nitrocellulose membranes (GE Healthcare). Membranes were blocked with 5% milk, incubated with primary antibodies in PBS with 0.1% Tween-20 (PBS-T) overnight at 4 °C, washed 4 times with PBS-T and incubated for 2 h with HRP-conjugated, secondary antibodies (Santa Cruz Biotechnology). Membranes were again washed 4 times, visualized using the ECL prime western blotting detection reagent (GE Healthcare) and imaged using a LAS-3000 imaging system (Fujifilm).

### Traction Force Microscopy

Polyacrylamide hydrogels with elastic moduli of 6 kPa or 12 kPa were prepared according to Fischer *et al*.^108^. Fluorescent beads (0.1 μm FluoSpheres, carboxylate-modified, LifeTechnologies) were encapsulated in the hydrogels to serve as displacement markers. FN or VN (100 μg/ml in PBS) were cross-linked to the surface using sulfosuccinimidyl 6-(4′-azido-2′-nitrophenylamino)hexanoate (sulfo-SANPAH; 2 mM, Thermo Fischer Scientific). REF_YFP-PAX_ were seeded on hydrogels and incubated for 6 hours prior to analysis on an upright microscope (Leica DM6000 B) equipped with a heating stage. Bead displacement fields were obtained by comparing images before and after cell removal using trypsin and employing particle image velocimetry using ImageJ^109^. Traction forces were calculated with a regularized Fourier transform traction cytometry (FTTC) algorithm written by Sabass *et al*.^110^ and summed up under the area of each cell.

### Statistical analysis & Graph plotting

Statistical analyses were performed using Prism software (GraphPad Inc.). Experimental data were analyzed using either unpaired t-tests or one-way ANOVA with Tukey post-test analysis, unless otherwise noted. The middle line in box plots indicates the median, the box indicates the interquartile range, the whiskers the 5^th^ and 95^th^ percentiles and the cross the mean. Black lines in dot plot represent mean values and error bars SEM. Column plots represent mean values and SEM. ‘n.s.’: not significant; *P < 0.05, **P < 0.01; ***P < 0.001; ****P < 0.0001.

## Additional Information

**How to cite this article**: Missirlis, D. *et al*. Substrate engagement of integrins α_5_β_1_ and α_v_β_3_ is necessary, but not sufficient, for high directional persistence in migration on fibronectin. *Sci. Rep*. **6**, 23258; doi: 10.1038/srep23258 (2016).

## Supplementary Material

Supplementary Information

Supplementary Video S1

Supplementary Video S2

Supplementary Video S3

Supplementary Video S4

Supplementary Video S5

Supplementary Video S6

Supplementary Video S7

Supplementary Video S8

## Figures and Tables

**Table 1:** Percentage of REF_WT_ that exhibit polarized protrusions and their mean directionality index (DI) from single cell motility assays on different coatings and under different experimental conditions.

Substrate	Treatment	n^1^ (N_exp_^2^)	% cells with polarized protrusions	DI
FN (10 μg/ml)	–	210 (4)	61	0.63
FN (1 μg/ml)	–	168 (3)	49	0.28
VN (10 μg/ml)	–	61 (2)	0	0.23
FN (10 μg/ml)	Serum-free	108 (2)	0	–
FN (10 μg/ml)	DMSO 0.1%	123 (2)	36	0.56
FN (10 μg/ml)	Soluble α_5_β_1_ antagonist	69 (2)	0	0.41
FN (10 μg/ml)	Soluble α_v_β_3_ antagonist	131 (2)	71	0.41
Patterned α_5_β_1_	–	41(2)	0	0.23
Patterned α_5_β_1_ & α_v_β_3_	–	75(2)	0	0.18

^1^n: number of analyzed cells.

^2^N_exp_: number of independent experiments.
