# Supplementary material for: Substrate engagement of integrins α5β1 and αvβ3 is necessary, but not sufficient, for high directional persistence in migration on fibronectin
Source: Sci Rep. 2016 Mar 18;6:23258. doi: 10.1038/srep23258 (PMC4796868; doi:10.1038/srep23258)
Supplement: Supplementary Information [file srep23258-s1.pdf]

Supplementary Information for article:

Substrate engagement of integrins $\alpha_5\beta_1$ and $\alpha_v\beta_3$ is necessary, but not sufficient, for high directional persistence in migration on fibronectin.

Dimitris Missirlis[1,*], Tamás Haraszti[1], Catharina v.C. Scheele[1], Tina Wiegand[1], Carolina Diaz[1], Stefanie Neubauer[2], Florian Rechenmacher[2], Horst Kessler[2], Joachim P. Spatz[1]

DMSO impairs polarized protrusion formation and directional persistence

DMSO had a negative effect on formation of polarized protrusions. The percentage of REF_{WT} that exhibited polarized protrusions on FN was 36% (44/123; N=2) and 22% (28/128; N=3) when DMSO was present at concentrations of 0.1% and 1%, respectively. Nevertheless, REF_{WT} remained elongated with stress fibers oriented along the major cell axis (Supplementary Fig. S7). Monitoring of adhesions using time-lapse TIRF microscopy upon addition of 1% DMSO revealed rapid NA disassembly, followed by polarized protrusion retraction and subsequent edge advancement without NAs (Video 8). The effects were less pronounced, but still evident at lower DMSO concentrations (Video 8). Importantly, directional persistence in fibroblast migration was significantly reduced in presence of 1% DMSO (Supplementary Fig. S7). The above data demonstrate significant effects of DMSO when it is present in the culture media during cell adhesion and migration studies, and call attention to the use of appropriate controls.

Scheme S1. Molecular structure of small-molecule, peptidomimetic, integrin antagonists that were used in our study. The antagonists were designed to selectively bind $\alpha_5\beta_1$ or $\alpha_v\beta_3$, while having a low affinity for $\alpha_{II}\beta_3$. Thiol-functionalized antagonists were synthesized to allow attachment to gold and surface patterning.

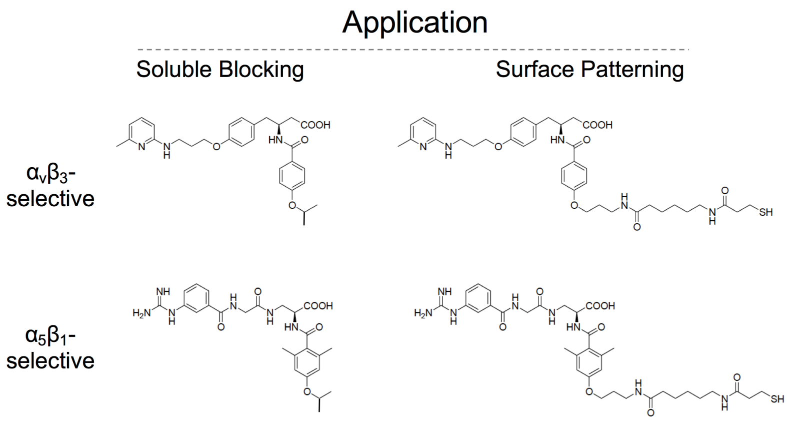

Table S1. List of antibodies against α_v or β_3 integrins that were tested with immunofluorescence microscopy with REF_{wt} or $REF_{YFP\text{-}PAX}$. None of these antibodies efficiently stained integrins on PFA- or methanol-fixed cells using standard protocols.

Antibody against	*Clone*	*Company*	*Cat. No.*
α_v integrin	H-2	Santa Cruz Biotechnology	sc-376156
α_v integrin	21/CD51	BD	611013
β_3 integrin	B3A	Chemicon	MAB2023Z
β_3 integrin	EPR2417Y	AbCam	ab75872

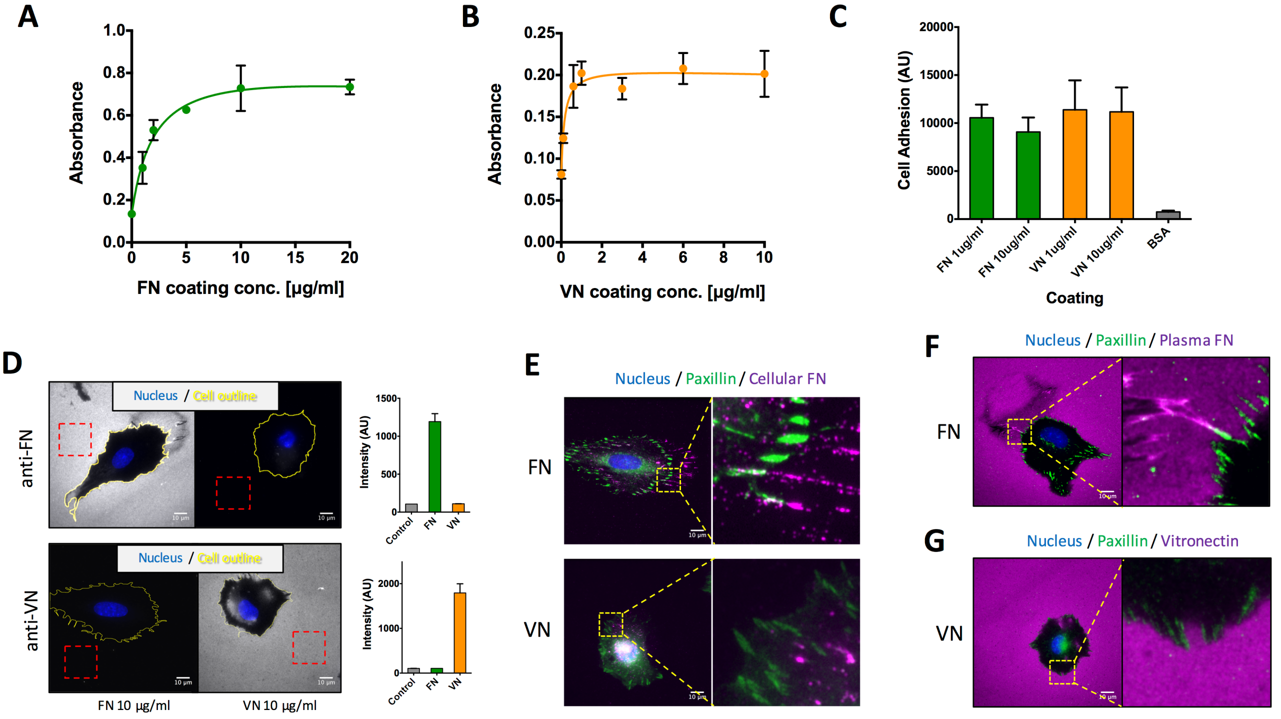

Figure S1. Fibroblasts adhere on FN and VN with similar efficiency, and remodel FN, but not VN coatings. Coating efficiency of adsorbed FN (**A**) and VN (**B**) on TCPS, detected by a modified ELISA assay. Surface saturation occurs at ≈10 µg/ml FN coating concentration and at ≈1 µg/ml VN coating concentration. Data are presented as mean±standard deviation (n=3) and fitted with the equation **y**=A***x**/(B+**x**)+C. (**C**) Relative efficiency of REF_{WT} adhesion on TCPS coated with FN or VN at 1 or 10 µg/ml. Adhesion was similar between coatings at the two different coating concentrations and much higher than adhesion on bovine serum albumin (BSA) coated substrates as the negative control. REF_{WT} were incubated for 20 minutes with indicated substrates and their numbers quantified using the Cyquant cell proliferation assay. Data are presented as mean±standard deviation (n=4; n=8 for BSA). (**D**) REF_{WT} were seeded on FN- or VN-coated substrates for 6 hours (10 µg/ml coating concentration) and stained against FN or VN. Fluorescence intensity from normalized images, acquired under identical settings, showed the absence of FN adsorption on VN-coated substrates and the absence of VN adsorption on FN-coated substrates. Quantification was performed from at least 3 different regions of the substrates (mean±standard deviation are presented). Control samples were not incubated with primary antibodies. Note that cells were not permeabilized and therefore dark regions under cells are due to antibody inaccessibility. (**E**) $REF_{YFP\text{-}PAX}$ cultured on FN- or VN-coated substrates for 6 hours (10 µg/ml coating concentration) and stained against cellular FN revealed formation of FN fibers deposited on FN, but limited fiber formation under the cells on VN and no deposition on the substrate. (**F**) Immunofluorescence staining against FN on FN-coated substrates or (**G**) against VN on VN-coated substrates revealed $REF_{YFP\text{-}PAX}$-induced remodeling in the case of FN coatings, but not VN coatings. Images were acquired 6 hours after seeding.

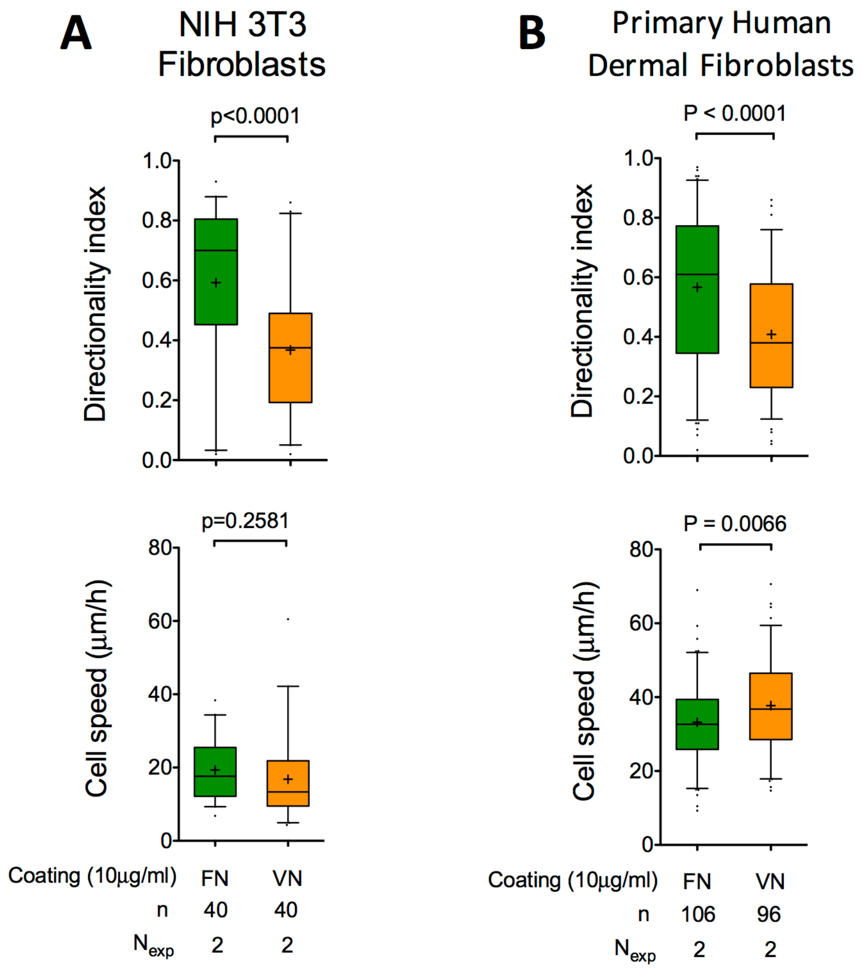

Figure S2. NIH3T3 and primary human dermal fibroblasts exhibit higher directional persistence on FN compared to VN. Cell speed and DI calculated for NIH-3T3 fibroblasts (**A**) and primary human dermal fibroblasts (**B**) from single cell migration studies on FN- or VN-coated TCPS (10 µg/ml). Experimental data were analyzed using an unpaired t-test. The middle line in box plots indicates the median, the box indicates the interquartile range, the whiskers the 5th and 95th percentiles and the cross the mean. n: number of analyzed cells; N_{exp}: number of independent experiments.

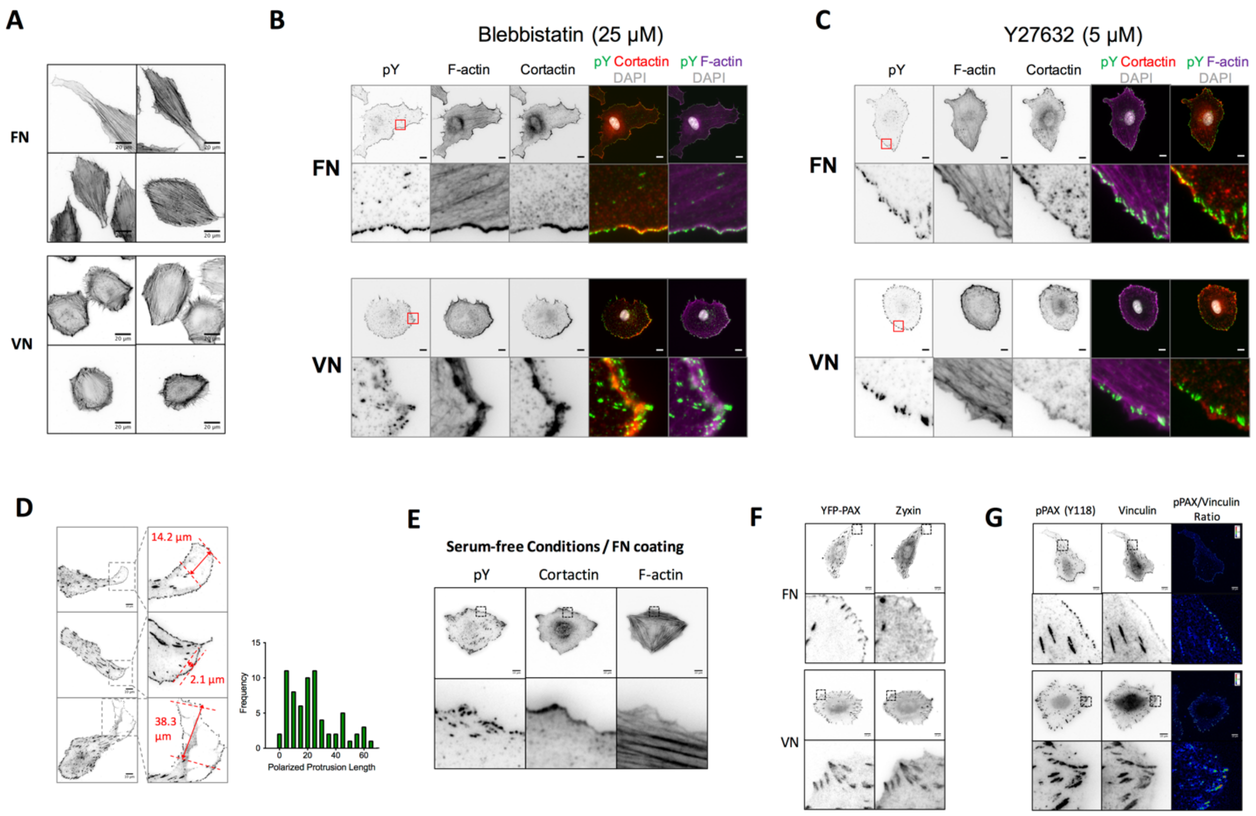

Figure S3. REF_{WT} adhesion on FN promotes formation of ventral stress fibers and polarized protrusions with nascent adhesions on their edge. (**A**) Images of REF_{WT} cultured for 6 hours on FN- or VN-coated glass (10 µg/ml coating concentration), in presence of 10% serum, fixed and stained against filamentous actin (F-actin) show coating-dependent stress fiber formation. (**B**) Myosin II or (**C**) Rho-associated protein kinase (ROCK) inhibition hinders FA and stress fiber assembly but not NA formation. REF_{WT} cultured for 5 hours on FN- or VN-coated substrates were incubated for 1 hour with the 25 µM blebbistatin (**B**) or 5 µM Y27632 (**C**), fixed and stained against F-actin, pY and cortactin. Scale bars: 10 µm. (**D**) Immunofluorescence microscopy images of pY-stained REF_{WT} were used to identify fibroblasts with polarized protrusions and quantify the distance between the cell edge and the first proximal elongated focal adhesion as shown in the examples presented. A histogram of polarized protrusion length for REF_{WT} cultured on FN-coated glass at a coating concentration of 10 µg/ml is shown (n=100 cells from 3 independent experiments). (**E**) Serum was required for polarized protrusion formation on FN, but not for NA formation or cortactin recruitment at the cell edge. REF_{WT} were cultured for 6 hours on FN- or VN-coated surfaces in the absence of serum, fixed and stained against F-actin, pY and cortactin. (**F**) Zyxin was present in FAs on both coatings but not in NAs on FN, in accordance with the requirement of tension for its recruitment. (**G**) Vinculin levels were lower on NAs compared to FAs on FN as shown through ratio imaging. (**F**) $REF_{YFP\text{-}PAX}$ or (**G**) REF_{WT} were cultured for 6 hours on FN- or VN-coated glass (10 µg/ml coating concentration), in presence of 10% serum, fixed and stained against zyxin or vinculin.

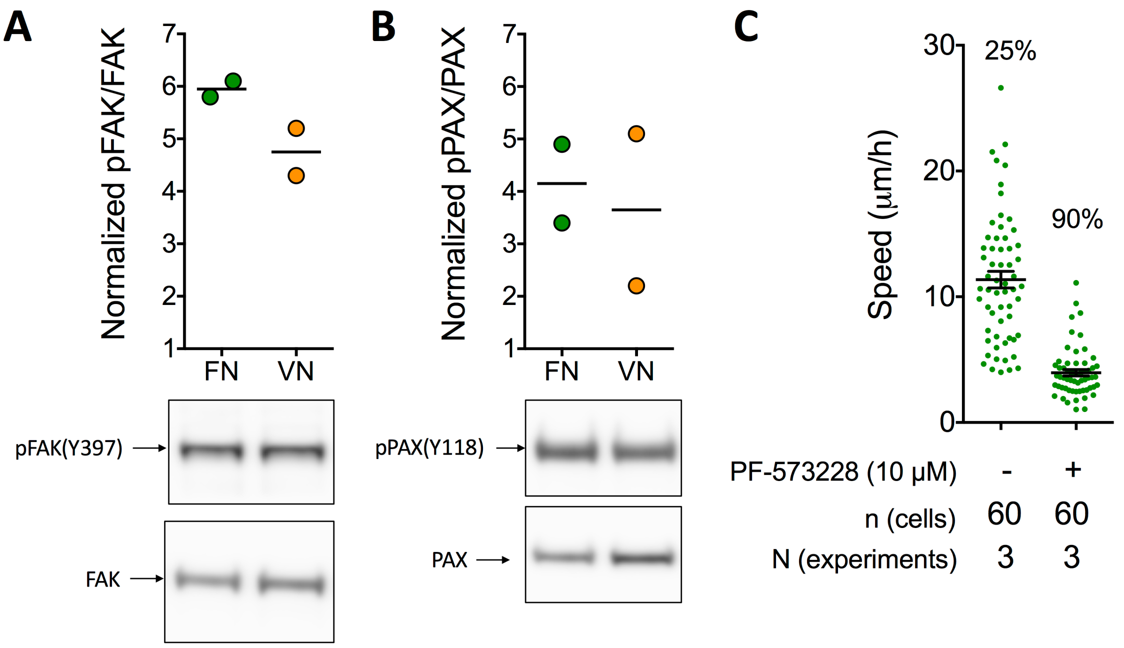

Figure S4. (**A**) FAK and (**B**) paxillin phosphorylation levels are similar in REF_{WT} cultured for 6 hours on FN versus VN (10 μg/ml coating concentration) as determined by western blot analysis. (**C**) FAK inhibition using the small molecule kinase inhibitor PF-573228 (10 μM) resulted in a pronounced decrease in cell speed and an increase in percentage of immotile cells (presented on the graph), compared to vehicle-only control conditions (DMSO 0.05% v/v). Dot plots with mean (**A**,**B**) or mean±SEM (**C**) are presented.

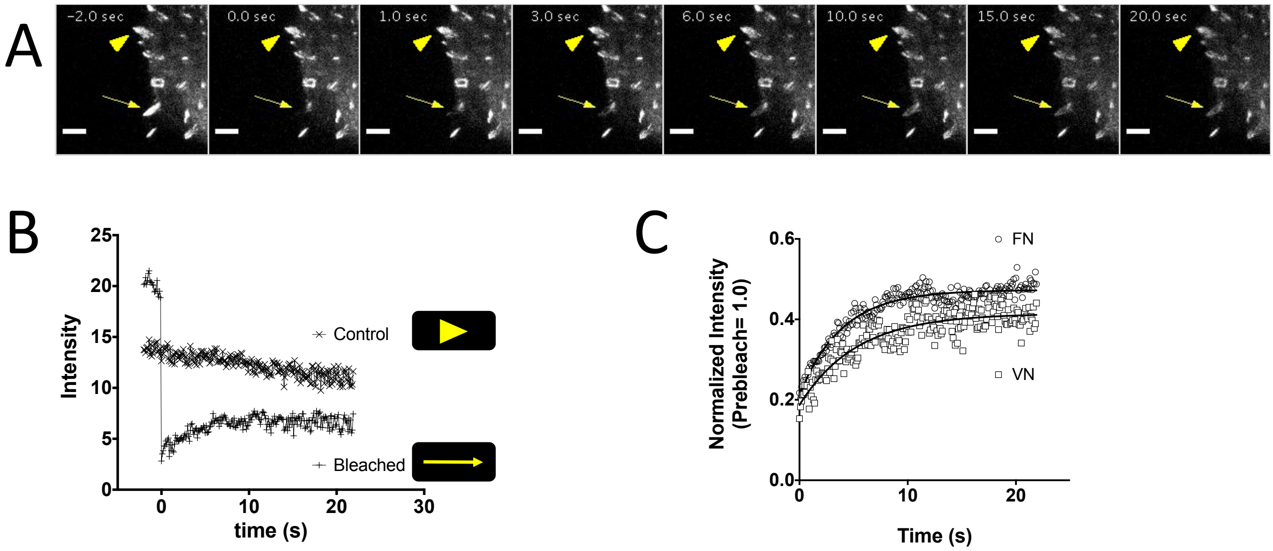

Figure S5. Typical results from a FRAP experiment. (**A**) Selected frames from a FRAP experiment with REF$_{YFP\text{-}PAX}$ on VN (10 μg/ml). The FA indicated by the yellow arrow was photo-bleached using a 488nm laser pulse. (**B**) Fluorescence intensity values of the photo-bleached FA (yellow arrow) and a control FA (yellow arrowhead) as a function of time. (**C**) Typical fluorescence recovery curves for FAs of REF$_{YFP\text{-}PAX}$ on VN or FN (10 μg/ml). Fluorescence intensity was normalized to the intensity before bleaching and corrected for non-specific bleaching due to imaging. The equation $y=y_0+(y_p-y_0)*(1-\exp(-kt))$ was used to fit the experimental data and extract recovery half time ($\ln(2)/k$) and mobile fraction (y_p-y_0).

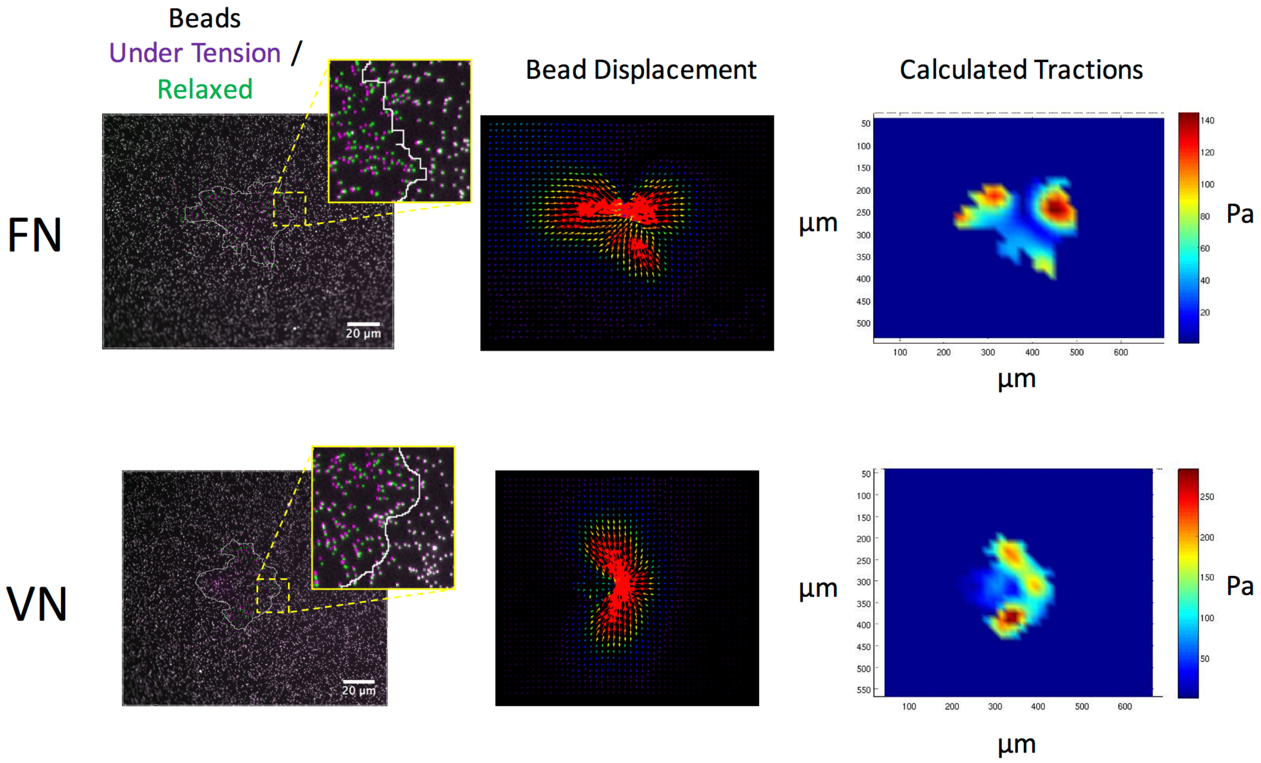

Figure S6. Typical traction force microscopy analysis and results for REF$_{YFP\text{-}PAX}$ cultured on FN- and VN-coated polyacrylamide gels with Young's moduli of 6 kPa. (**A**) Overlay of epifluorescence microscopy images of fluorescent beads before (magenta) and after (green) cell removal by trypsin. Cell outlines are shown as white lines. (**B**) Bead displacement fields were calculated by comparing images before and after cell removal using a particle image velocimetry plugin in ImageJ. (**C**) Traction forces were calculated with a regularized Fourier transform traction cytometry (FTTC) algorithm .

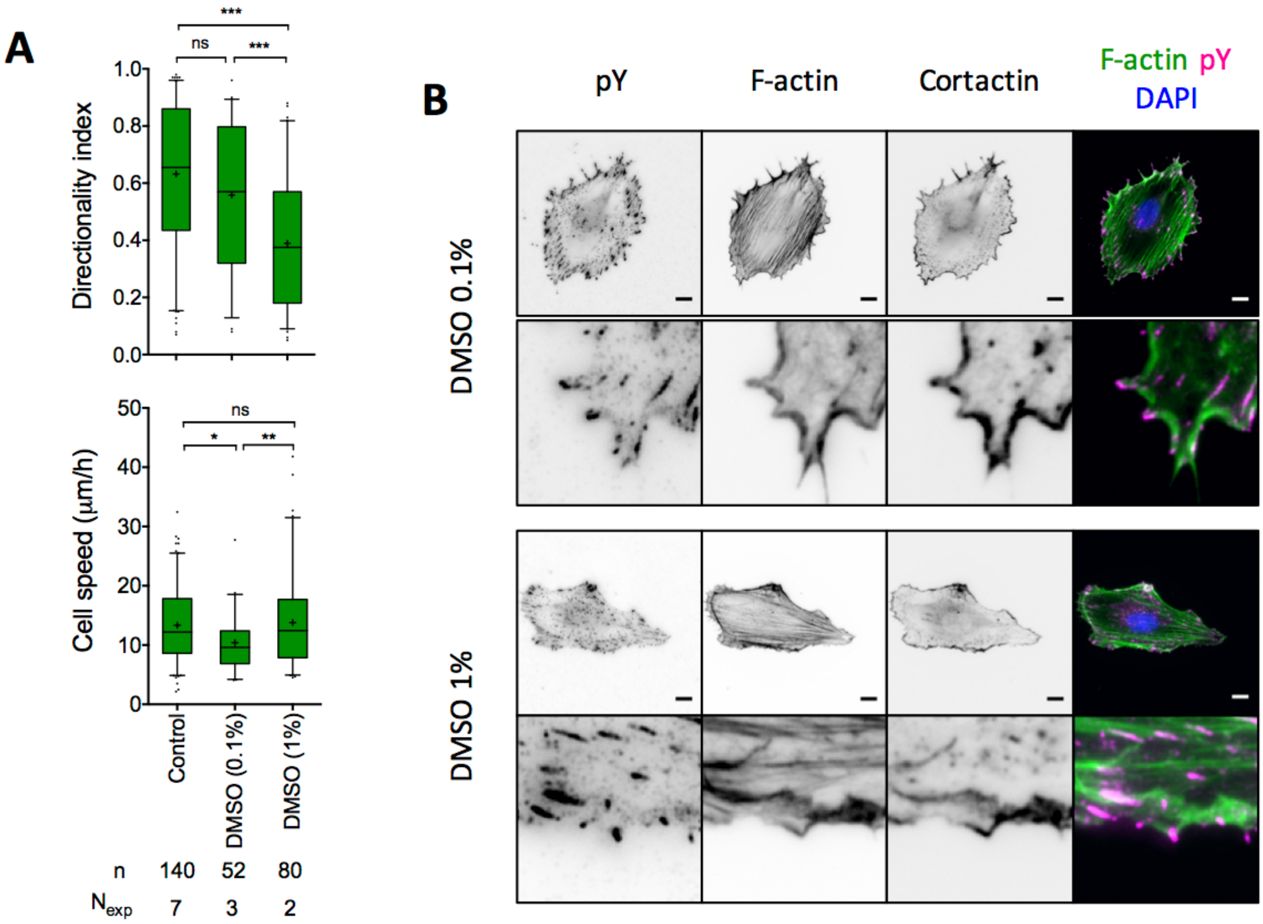

Figure S7. DMSO inhibits polarized protrusion formation and has a negative effect on directional persistence at high concentrations. (**A**) REF_{WT} cell speed and DI on FN (10 µg/ml) in presence of different 0.1% or 1% DMSO. Data for control conditions (FN) and for 0.1% DMSO are included for clarity and are the same as in Figures 1 and 5 of the main text, respectively. Low DMSO concentration does not significantly affect REF_{WT} DI and causes a slight reduction in average cell speed. Presence of higher DMSO concentration (1%) impairs directional migration, even though it has no effect on cell speed. (**B**) Representative immunofluorescence images of REF_{WT} cultured for 5 hours on FN (10 µg/ml) and 1 hour in presence of DMSO prior to fixation. Formation of polarized protrusions and NAs was impaired, but polarized cytoskeleton and ventral stress fiber formation was similar to that in control cells on FN. n: number of analyzed cells; N_{exp}: number of independent experiments; Scale bars: 10 µm. Data were compared using one-way ANOVA with Tukey post-test analysis (n.s.: not significant; * $p<0.05$, ** $p<0.01$, *** $p<0.001$).

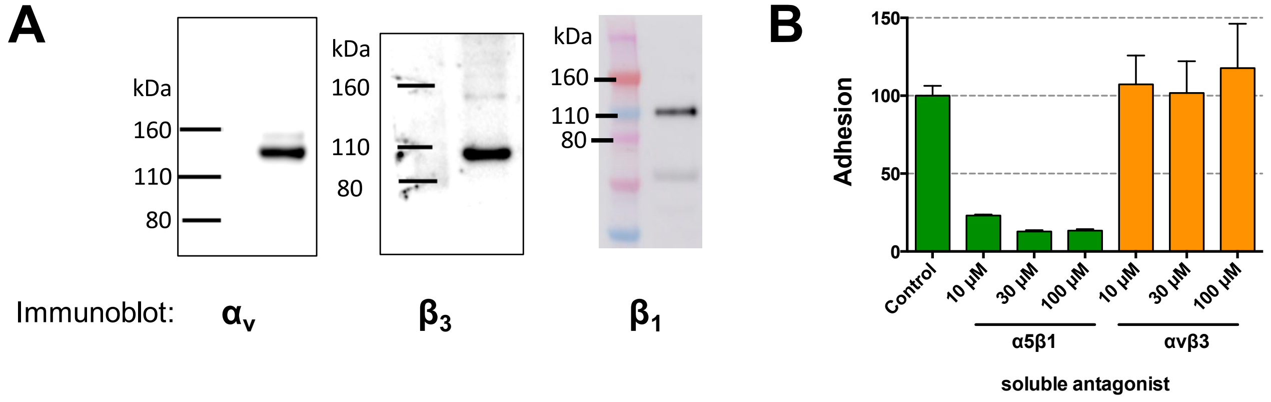

Figure S8. REF_{WT} utilize mainly $\alpha_5\beta_1$ integrins to initially adhere to FN. (**A**) Western blots of REF_{WT} lysates, confirming the expression of α_V, β_3 and β_1 integrins. (**B**) The percentage of REF_{WT} that adhered to FN-coated substrates (1 µg/ml coating concentration) in presence of selective integrin antagonists against $\alpha_5\beta_1$ or $\alpha_v\beta_3$ are shown. Data are normalized to control conditions in the absence of integrin antagonists. $\alpha_5\beta_1$ integrin antagonists inhibited fibroblast adhesion on FN, whereas $\alpha_v\beta_3$ had no effect in this respect. Results are presented as the mean ± standard deviation (n=3).

Video Legends

Video 1. Fibroblasts exhibit directionally persisten migration on fibronectin and random migration on vitronectin. Time-lapse, phase contrast imaging of REF_{WT} migrating on plastic dishes coated with 10 µg/ml FN (left) or 10 µg/ml VN (right). First frame (t=00:00) was captured 4 hours after cell seeding. Time is displayed in hour:min format.

Video 2. Integrin α5 clusters in adhesions on FN but not VN. Time-lapse, TIRF imaging of REF_{WT} transfected with α5-GFP plasmid migrating on glass coated with 10 µg/ml FN or VN. Zoomed-in regions are displayed on the left panels. First frame (t=00:00) was captured approximately 1 hour after cell seeding. Time is displayed in hour:min format.

Video 3. Integrin β3 clusters in adhesion on both FN and VN. Time-lapse, TIRF imaging of REF_{WT} transfected with β3-YFP plasmid migrating on glass coated with 10 µg/ml FN or VN. Zoomed-in regions are displayed on the left panels. First frame (t=00:00) was captured approximately 1.5 hours after cell seeding. Time is displayed in hour:min format. Scale bars: 10 µm.

Video 4. Fibroblasts exhibit sliding trailing FAs on FN and assemble adhesions at sites of older, disassembled adhesions on VN. Time-lapse, TIRF imaging of $REF_{YFP-PAX}$ migrating on glass coated with 10 μg/ml FN (left) or VN (right). Zoomed-in regions of locations where FA disassemble and subsequently re-assemble, as the cell is moving around, are shown for the VN-coated surface. First frame was captured approximately 6 hours after cell seeding. Scale bars: 10 μm.

Video 5. Polarized protrusions exhibiting NAs are formed at the leading edge of fibroblasts on FN. Time-lapse, TIRF imaging of $REF_{YFP-PAX}$ migrating on glass coated with 10 μg/ml FN (left) or VN (right). New adhesions formed preferentially at the leading edge on FN, but assembled randomly around the cell periphery on VN. First frame (t=0) was captured approximately 4 hours after cell seeding. Scale bar: 10 μm.

Video 6. Lamellipodium protrusion and retraction cycles are localized at the leading edge of fibroblasts on FN. Time-lapse, phase contrast imaging of REF_{WT} seeded on glass coated with 10 μg/ml FN. Membrane protrusion activity is mostly confined at the leading edge of the migrating fibroblasts. First frame (t=00:00) was captured approximately 5 hours after cell seeding. Time is displayed in min:sec format.

Video 7. Lamellipodia are formed around the cell periphery on VN and are more dynamic compared to FN. Time-lapse, phase contrast imaging of REF_{WT} seeded on glass coated with 10 μg/ml VN. Membrane ruffling activity is observed around the cell edges and is more intense compared to FN. First frame (t=00:00) was captured approximately 4 hours after cell seeding. Time is displayed in min:sec format.

Video 8. DMSO results in disruption of NAs and polarized protrusions. TIRF imaging of $REF_{YFP-PAX}$ migrating on glass coated with 10 μg/ml FN. Time t=0 min corresponds to DMSO addition to the cell culture medium to achieve the indicated final concentration. First frame was captured approximately 4 hours after cell seeding. Zoomed-in regions are displayed on the left panels. Scale bars: 10 μm.
